# Accurate and fast segmentation of filaments and membranes in micrographs and tomograms with TARDIS

**DOI:** 10.1101/2024.12.19.629196

**Published:** 2024-12-20

**Authors:** Robert Kiewisz, Gunar Fabig, Will Conway, Jake Johnston, Victor A. Kostyuchenko, Cyril Bařinka, Oliver Clarke, Magdalena Magaj, Hossein Yazdkhasti, Francesca Vallese, Shee-Mei Lok, Stefanie Redemann, Thomas Müller-Reichert, Tristan Bepler

**Affiliations:** 1 Simons Machine Learning Center, New York Structural Biology Center, New York, United States; 2 Simons Electron Microscopy Center, New York Structural Biology Center, New York, United States; 3 Experimental Center, Faculty of Medicine Carl Gustav Carus, Technische Universität Dresden, Dresden, Germany; 4 Center for Computational Biology, Flatiron Institute, New York, United State; 5 Department of Physiology and Cellular Biophysics, Columbia University Irving Medical Center, New York, United States; 6 Programme in Emerging Infectious Diseases, Duke-National University of Singapore Medical School, Singapore, Singapore; 7 Centre for Bioimaging Sciences, Department of Biological Sciences, National University of Singapore, Singapore, Singapore; 8 Institute of Biotechnology of the Czech Academy of Sciences, BIOCEV, Vestec, Czech Republic; 9 Department of Anesthesiology, Columbia University Irving Medical Center, New York, United States; 10 Department of Cell Biology, University of Virginia School of Medicine, Charlottesville, United States; 11 Department of Molecular Physiology and Biological Physics, University of Virginia, School of Medicine, Charlottesville, United States; 12 Center for Membrane and Cell Physiology, University of Virginia School of Medicine, Charlottesville, United States; 13 Structural Biology Initiative, CUNY Advanced Science Research Center, New York, United States; 14 Department of Chemistry and Biochemistry, City College of New York, United States; 15 Core Facility Cellular Imaging, Faculty of Medicine Carl Gustav Carus, Technische Universität Dresden, Dresden, Germany

**Keywords:** Cryo-EM/ET, TEM EM/ET, Segmentation, TARDIS, CNN, DIST, Semantic Segmentation, Instance Segmentation, Filaments, Microtubules, Membranes, Point Cloud

## Abstract

It is now possible to generate large volumes of high-quality images of biomolecules at near-atomic resolution and in near-native states using cryogenic electron microscopy/electron tomography (Cryo-EM/ET). However, the precise annotation of structures like filaments and membranes remains a major barrier towards applying these methods in high-throughput. To address this, we present TARDIS (**T**ransformer-b**a**sed **R**apid **D**imensionless **I**nstance **S**egmentation), a machine-learning framework for fast and accurate annotation of micrographs and tomograms. TARDIS combines deep learning for semantic segmentation with a novel geometric model for precise instance segmentation of various macromolecules. We develop pre-trained models within TARDIS for segmenting microtubules and membranes, demonstrating high accuracy across multiple modalities and resolutions, enabling segmentation of over 13,000 tomograms from the CZI Cryo-Electron Tomography data portal. As a modular framework, TARDIS can be extended to new structures and imaging modalities with minimal modification. TARDIS is open-source and freely available at https://github.com/SMLC-NYSBC/TARDIS, and accelerates analysis of high-resolution biomolecular structural imaging data.

## Introduction

Electron microscopy^1,2^ (EM) and electron tomography^3–6^ (ET) are leading a revolution in structural biology by enabling high-resolution^7–12^ visualization of complex cellular and macromolecular structures^13–15^. Advancements in EM technology^16^, such as improved microscope optics, detectors, sample preparation techniques, and data collection automation, have significantly increased acquisition speed and quality. As a result, researchers can now collect tomograms rapidly, leading to the accumulation of tens of thousands of tomograms in databases like EMPIAR^17^ and the CZI data portal^18,19^. However, the analysis of these tomograms, especially the annotation of biological structures of interest, is now the primary barrier to unlocking the full potential of this data for studying biological systems. As an example, up to ~300 tomograms can be collected in a single day, but researchers currently spend months painstakingly annotating their biomolecules of interest for downstream analysis^20,21^. Large-scale imaging efforts across the scientific community are now generating thousands of tomograms annually. Notably, the Electron Microscopy Data Bank (EMDB) alone has recorded 8,640 tomogram deposits this year^22^. This surge in data generation requires rapid, automatic annotation methods to identify the position, volume, and quantity of cellular macromolecules^21^ to extract biological insights from these rich data sources.

In order to quantitatively study biomolecules, such as microtubules^10,23^, membranes^14^, ribosomes^24,25^, or viral particles^13,26^, thousands of tomograms must be analyzed to gain insights into structural dynamics and interactions^10,11,27–30^. Yet, studies remain limited to only a handful of datasets that can feasibly be annotated^7,21,31–33^. Large-scale statistical analyses and comparative studies across different conditions or time points, require tens to hundreds of annotated tomograms to unlock new insights in biophysical analysis. This is not possible with current annotation approaches. Therefore, faster and more efficient annotation methods are required to realize the promise of tomography for large scale biological studies.

Several tools have been developed to assist, simplify, or automate the annotation of membranes and microtubules in EM data, but they often fall short of meeting researchers needs. Traditional, general-purpose software like Fiji^34^, Napari^35^, and IMOD^36^ focus on manual annotation which is time consuming. Specialized tools such as TomoSegMemTV^37^ and MemBrain-seg V2^38^ for membrane segmentation, or Amira^39^ for microtubule segmentation, depend on high-quality tomograms and require extensive parameter tuning to achieve acceptable results. Moreover, these tools are designed for the annotation of a single type of macromolecule or work with one type of EM method. There are also tools such as Dragonfly^40^ which have been developed as general-purpose deep learning workflows that allow automatic annotation of any macromolecule. However, Dragonfly requires training on a case-by-case basis using labels manually provided by the user, necessitating significant labeling and correction effort, and is limited to the prediction of structures from the background at the voxel level. Dragonfly cannot be applied to problems where individual instances are required, which is usually the case for biomolecule analysis. Thus, fast, accurate, out-of-the-box algorithms that can be applied across diverse datasets for common biomolecules are critical.

Here, we present TARDIS (**T**r**a**nsformer-based **R**apid **D**imensionless **I**nstance **S**egmentation)^41^, a modular framework for annotating biomolecular structures both in EM micrographs and tomograms. TARDIS includes state-of-the-art pre-trained networks for membrane and microtubule annotation. To develop these pre-trained models, we created an extensively manually annotated dataset of membranes and microtubules across tomograms and micrographs obtained from plastic sections and cryo-EM/ET. In total, this dataset contains 382 images with 71,747 objects, the largest manually labeled dataset of its kind to date (Suppl. Table 1). As a flexible framework, TARDIS is straightforward to extend with semantic segmentation networks for new macromolecules. These integrate seamlessly with our pre-trained instance segmentation network to automatically identify object instances from the semantic maps, enabled by our novel point cloud-based instance segmentation model architecture, **D**imensionless **I**nstance **S**egmentation **T**ransformer (DIST)^42^, an SO(n) equivariant transformer.

We demonstrate that TARDIS accurately annotates filaments and membranes. TARDIS improves annotation accuracy by 42% and 55% over existing tools for microtubules and membranes respectively. TARDIS is also fast, annotating a single tomogram in a matter of minutes, orders of magnitude faster than manual annotation, and on average 2x faster than other algorithms for membrane and 5x faster for microtubule segmentation. As a pluggable framework, TARDIS integrates seamlessly with other tools for semantic segmentation to provide instance predictions and is adaptable to new macromolecules and data types. We demonstrate that TARDIS can be applied without any re-training to segmentation of tobacco mosaic virus (TMV)^43,44^ in cryo-ET. We also build and plugin a new semantic segmentation network for actin in cryo-ET and deploy a semantic segmentation network for microtubules in fluorescent microscopy images all within the TARDIS framework. Using TARDIS, we generated the largest, high-quality annotation of microtubules and membranes across 13,000 tomograms - a task that would have taken an experienced human annotator approximately 35 years to complete manually. These annotations are available to the community in the CZI Cryo-ET data portal^18,19^.

TARDIS is open-source and freely available at https://github.com/SMLC-NYSBC/TARDIS, and we also provide an optional Napari plugin-based GUI for TARDIS at https://github.com/SMLC-NYSBC/napari-tardis_em.

## Results

### TARDIS is a modular framework for any macromolecular segmentation

**Figure 1:**
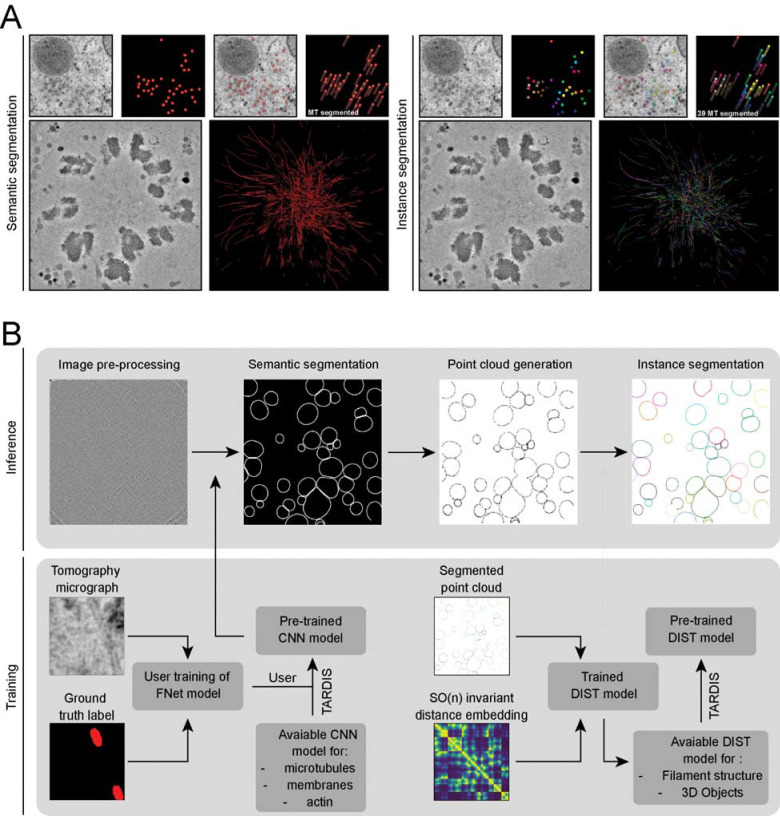
Overview of the TARDIS framework for semantic and instance segmentation **A)** Example illustrating the distinction between semantic and instance segmentation. **B)** Segmentation of micrographs or tomograms begins with a CNN. TARDIS uses custom pre-trained CNN models to predict actin, membrane or microtubules from tomograms, micrographs and TIRF images. Predicted semantic masks provide the initial segmentation of structures. These masks are then skeletonized to produce a point cloud representing the core structure of each object. Instance segmentation is finalized using the DIST model, which predicts connection probabilities between points in the point cloud. The final instance prediction is obtained through a greedy graph cut.

TARDIS is a framework for fully automated semantic and instance segmentation of cellular macromolecules across diverse resolutions and imaging modalities (Fig. 1A). It surpasses existing tools by offering broad applicability without requiring extensive manual parameter tuning. Optimized for accuracy and speed, TARDIS enables rapid annotation of large datasets. Its modular architecture supports seamless extensions to incorporate new macromolecular types (Fig. 1B).

The framework leverages pre-trained networks for 2D and 3D segmentation tasks and currently includes specialized semantic segmentation models for the following sub-cellular features:

- Microtubules in 3D electron tomograms (including cryo and plastic section)
- Microtubules in 2D total internal reflection fluorescence (TIRF) images
- Membranes in 2D cryo-electron micrographs
- Membranes in 3D cryo-electron tomograms
- Actin filaments in 3D cryo-electron tomograms

By combining these networks with our novel pre-trained point cloud-based instance segmentation model (DIST), TARDIS accurately distinguishes individual structures, enabling quantitative analyses across thousands of tomograms. We provide pre-trained DIST models for two general structures: linear structures (DIST-linear), e.g., filaments or membranes in 2D, and surfaces (DIST-surface), e.g., membranes in 3D. We refer to these collectively as the pre-trained instance segmentation models but apply DIST-linear to filaments and other linear objects and DIST-surface to 3D surfaces when applicable.

### High-Accuracy Semantic Segmentation with FNet

This framework is anchored by our custom-designed FNet, a CNN model for semantic segmentation (Suppl. Fig. 1; Methods). FNet, a variant of the UNet architecture, provides improved semantic segmentation performance by combining a single UNet-style encoder with two concurrent decoders. One with conventional skip-connections and another with fully connected skip-connections. This architecture improves the precision of cellular structure segmentation, generating probability maps that are converted to binary masks and processed into point clouds (Suppl. Fig. 2A–B).

### Geometry-aware instance segmentation with DIST

Point clouds are extracted from voxel-level probability maps through skeletonization, which retrieves the skeletons of segmented structures (Suppl. Fig. 2A; Methods). The DIST model predicts graph edges between points to delineate individual object instances. Operating on point clouds of any dimensionality, the model utilizes an SO(n) equivariant representation and integrates geometry-aware operations within a transformer network (Suppl. Fig. 3; Methods). Instances are identified by applying a graph-cut to the edge probability outputs, isolating the most likely connected components as distinct instances.

### TARDIS accurately segments microtubule and membrane instances in tomograms

TARDIS aims to alleviate the bottleneck in annotating large tomographic data. To validate its performance, we benchmarked TARDIS against existing tools, such as Amira, MemBrain-seg V2 and TomoSegMemTV, focusing on microtubule and membrane segmentation across datasets of varying quality and resolution (Suppl. Table 1). Due to the lack of suitable public data, we created a benchmark dataset comprising hand-curated microtubules and membranes. Specifically, we fully instance-labeled membranes in 120 micrographs and 53 tomograms and microtubules in 91 tomograms and 112 TIRF images (Methods).

Semantic segmentation is evaluated using F1 score, precision, recall, average precision (AP), and precision at 90% recall (P90). We evaluated instance segmentation using the mean coverage (mCov) score, which quantifies the overlap between ground truth and predicted point clouds. These metrics range from 0 to 1, with higher values indicating better performance (Methods).

### 3D microtubules segmentation

**Figure 2:**
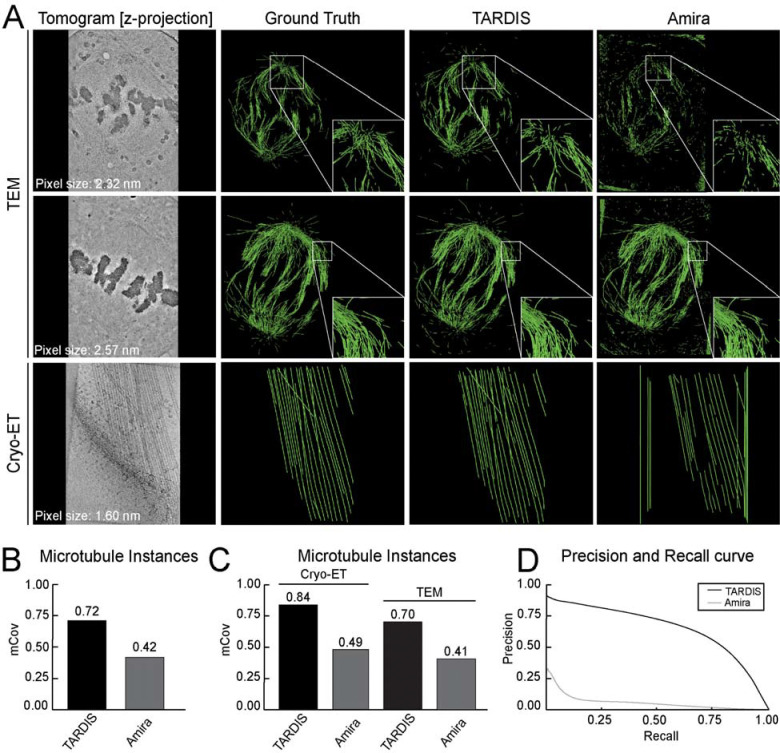
Microtubule segmentation examples and comparison with other state-of-the-art methods **A)** Instance segmentation examples from TARDIS and Amira, with green lines indicating renders of the core of microtubule filament instances. **B)** mCov metric comparing instance segmentation results from Amira and TARDIS on an entire benchmark dataset. **C)** mCov metric showing instance segmentation results for microtubules using TARDIS and Amira on benchmark datasets from Cryo-ET and plastic ET reconstructed volumes. **D)** Precision-recall curves illustrating the quality of semantic segmentation between Amira and TARDIS.

We evaluated TARDIS on microtubule segmentation task using dataset from both Cryo-ET and plastic ET, with resolutions ranging from 16.00 to 25.72 Å per pixel (Fig. 2A and Suppl. Table 1). Validation results confirmed that the DIST-linear model significantly improves instance segmentation of structures using point clouds, achieving near-perfect scores (Suppl. Table 2). Benchmarking on curated datasets further demonstrated TARDIS improvements over Amira, with TARDIS achieving an mCov score of 0.72 compared to Amira 0.42 (Fig. 2B). These results underscore TARDIS higher segmentation accuracy, particularly in high-resolution tomograms (≤18 Å), where Amira performance declined (Fig. 2C and Suppl. Fig. 4A).

For semantic segmentation, TARDIS consistently achieved a higher F1 score (0.61 vs. 0.17 for Amira), indicating better retrieval of microtubules with significantly fewer false positives. At high recall levels, TARDIS maintained a precision of 0.33, while Amira precision dropped to 0.01 at 90% recall (Fig. 2D). Although the Amira template-matching algorithm was previously applied on the plastic ET dataset, its lower accuracy necessitated substantial manual corrections^10,11,27^. In contrast, TARDIS consistently segmented more microtubules with significantly less manual adjustment (Fig. 2A, Suppl. Fig. 4A and Table 1).

### 3D membrane segmentation

**Figure 3:**
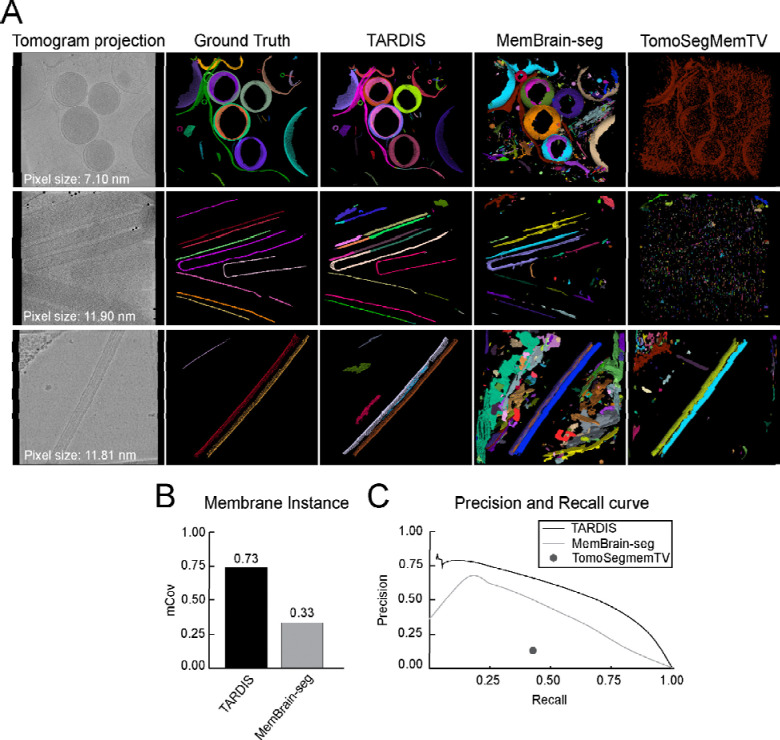
Membrane segmentation from tomographic data examples and comparison with other state-of-the-art methods **A)** Instance segmentation examples from TARDIS, MemBrain-seg V2, and TomoSegMemTV, with multicolor renderings indicating individual membrane instances. **C)** mCov metric showing a comparison of instance segmentation results between TARDIS and MemBrain-seg V2. TomoSegMemTV was excluded from the comparison as it achieved results close to 0 due to many datasets segmented only as a single instance. **C)** Precision-recall curves illustrating the quality of semantic segmentation between TARDIS, MemBrain-seg V2, and TomoSegMemTV.

We assessed TARDIS for membrane segmentation across varying tomogram resolutions and membrane origins (Fig. 3A and Suppl. Table 1). For membrane instance segmentation, we compared TARDIS exclusively with MemBrain-seg V2, which uses a connected component algorithm. TomoSegMemTV was excluded from this analysis due to its frequent generation of single-instance segmentations, making it unsuitable for detailed analyses.

We validated that the DIST-surface model improved instance segmentation of surface-like structures using point clouds achieving an almost perfect mCov score of 0.91 (Suppl. Table 2). Benchmarking on tomographic datasets further demonstrated TARDIS performance, achieving an mCov score of 0.73, more than doubling the performance of MemBrain-seg V2, which scored 0.33 (Fig. 3B). This underscores TARDIS ability to accurately segment membrane instances. Notably, applying the DIST model to membranes initially segmented with MemBrain-seg V2 significantly improved the mCov score from 0.33 to 0.68 (Suppl. Fig. 6 A–B).

Beyond instance segmentation, TARDIS significantly improves semantic segmentation through the incorporation of the FNet architecture. We evaluated all methods on semantic segmentation tasks, where TARDIS consistently achieved the highest F1 scores (Table 2). Although TARDIS had a lower recall rate of 0.65 compared to 0.72 from MemBrain-seg V2, it demonstrated superior precision-recall trade-offs across various decision thresholds (Fig. 3C, Suppl. Fig. 4B, and Table 2). To further enhance membrane segmentation, we implemented a pixel size normalization strategy. Scaling tomograms to an optimal resolution of 15 Å and micrographs to 4 Å yielded substantial improvements (Suppl. Results). Specifically, the FNet model scaled to 4 Å achieved the best results for high-resolution micrographs (<10 Å), while scaling to 8 Å provided the highest accuracy for low-resolution micrographs. Based on these findings, we integrated the optimal pixel size normalization into the automated workflow and publicly released the enhanced model, providing the community with immediate access to this improved tool.

### Extending TARDIS to new biological structures and imaging modalities

TARDIS demonstrates high accuracy and efficiency in segmenting microtubules and membranes in electron microscopy datasets, but its versatility extends well beyond these applications. Designed as a modular segmentation tool, TARDIS handles diverse macromolecular structures. To assess this, we evaluated TARDIS on various biological structures and imaging modalities, showing its capacity to generalize across different datasets with minimal or no retraining. We assessed TARDIS ability to accurately segment 2D membranes, actin filaments, and the Tobacco Mosaic Virus (TMV). Given the lack of alternative tools capable of performing instance segmentation for these structures, we identified a distance-based grouping method as a comparison. This method is analogous to the connected component algorithm used by MemBrain-seg V2. All methods were benchmarked using a dataset annotated by an experienced user to assess their effectiveness.

### 2D membrane segmentation

**Figure 4:**
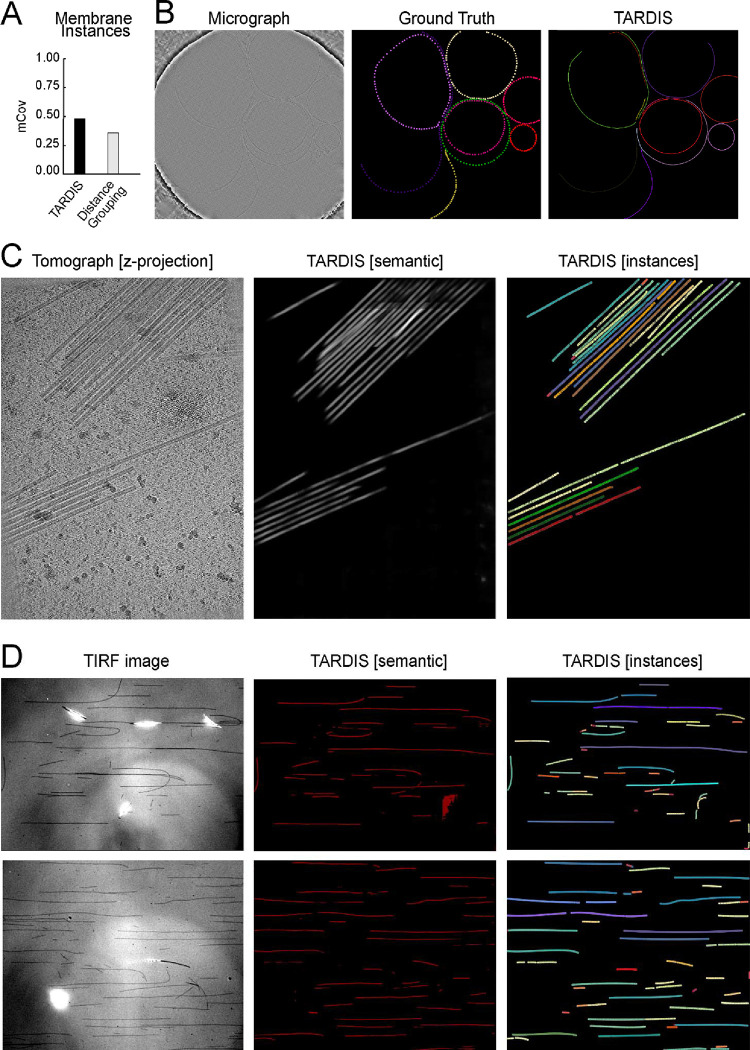
Example of TARDIS applications where one of the models was reused, showcasing TARDIS generalizability **A)** TARDIS benchmark results on instance segmentation task compared to distance grouping method. **B)** Example of TARDIS instance segmentation of membranes in 2D using the pre-train 2D membrane semantic segmentation model and DIST-linear. **C)** Examples of TARDIS instance and semantic segmentation of Tobacco Mosaic Virus (TMV) filaments, achieved by fully reusing the TARDIS microtubule segmentation workflow. **D)** Examples of TARDIS instance and semantic segmentation of stabilized microtubules from IRM images using TARDIS pre-train FNet model and re-used DIST-linear model.

We evaluate TARDIS performance on 2D membrane segmentation by training the FNet model for semantic segmentation on a manually annotated dataset. We also incorporated ~60 nm thick tomogram slices projected into 2D, which we observed to improve model performance (Suppl. Table 3). For instance segmentation, we applied the pre-trained DIST-linear model. This approach allowed TARDIS to accurately identify individual membrane instances, achieving the highest mCov score among all tested methods (Fig. 4A–B).

We tested the pre-trained TARDIS FNet model for semantic segmentation, where it outperformed a similarly deep UNet model, achieving superior F1 scores, particularly in high-resolution micrographs (≤10 Å). TARDIS achieved a precision of 0.74 for membrane structure recovery (Suppl. Fig. 5A–B; Table 3). Further analysis demonstrated that the TARDIS FNet model performed well across both high-resolution (≤10 Å) and low-resolution (>10 Å) datasets, achieving an F1 score of 0.41 on lower-resolution images while maintaining high precision (Suppl. Fig. 5C; Table 3).

In summary, the TARDIS FNet model has established a new benchmark for 2D membrane segmentation, demonstrating superior performance across various micrograph resolutions. Moreover, we demonstrated that the TARDIS instance segmentation pipeline can be easily reused. This achievement underscores TARDIS as the first open-source tool capable of reliably segmenting membranes from micrographs and accurately retrieving instances.

### Segmenting TMV in Cryo-ET without TARDIS re-training

To evaluate TARDIS adaptability to different filamentous structures, we applied it to segment the TMV in Cryo-ET datasets^45^. For this task, we did not retrain any models. Instead, we employed the pre-trained FNet model, originally developed for 3D microtubule segmentation, alongside the pre-trained DIST-linear model. Despite structural differences between microtubules and TMV, TARDIS successfully segmented TMV filaments without any fine-tuning (Fig. 4C). This demonstrates the framework ability to handle diverse filamentous entities with minimal manual intervention.

### TARDIS can segment actin filament trained in limited data scenarios

In many biological studies, limited availability of training data presents a significant challenge for deep learning-based models. To evaluate TARDIS performance under such constraints, we tested it on actin filament segmentation using three annotated tomograms. For comparison, both the TARDIS FNet and a standard UNet model were trained on this small dataset. Despite the limited data, TARDIS outperformed UNet across all metrics (Suppl. Table 4) and achieved superior instance recovery, with an mCov score of 0.12 compared to UNet 0.10. These results demonstrate TARDIS robustness and adaptability for novel segmentation tasks, even when training data are scarce.

### Fluorescence microscopy microtubule segmentation

While TARDIS demonstrates effectiveness on EM data, it is designed as a modular tool capable of addressing tasks beyond electron microscopy. To showcase its broader applicability, we tested TARDIS on non-EM images by training the FNet network on TIRF microscopy images of taxol-stabilized microtubules. Utilizing the pre-trained DIST-linear, TARDIS successfully segmented and recovered nearly all microtubule instances from the benchmark dataset (Fig. 4D). These highlight TARDIS modular design and its effectiveness in adapting to various imaging modalities while maintaining high performance in instance recovery and segmentation.

### TARDIS Enables Large-Scale Annotation for Biophysical Analysis

In electron microscopy, a significant challenge is the publication of thousands of tomograms without accompanying segmentation data. This gap often stems from researchers either lacking effective tools for semantic and instance segmentation or choosing not to perform segmentation. As a result, vast repositories of valuable datasets remain underutilized, with only a few research groups possessing the resources required for segmentation, limiting broader accessibility and analysis. TARDIS can fill this gap by enabling fast and accurate segmentation of biomolecules in these datasets.

**Figure 5:**
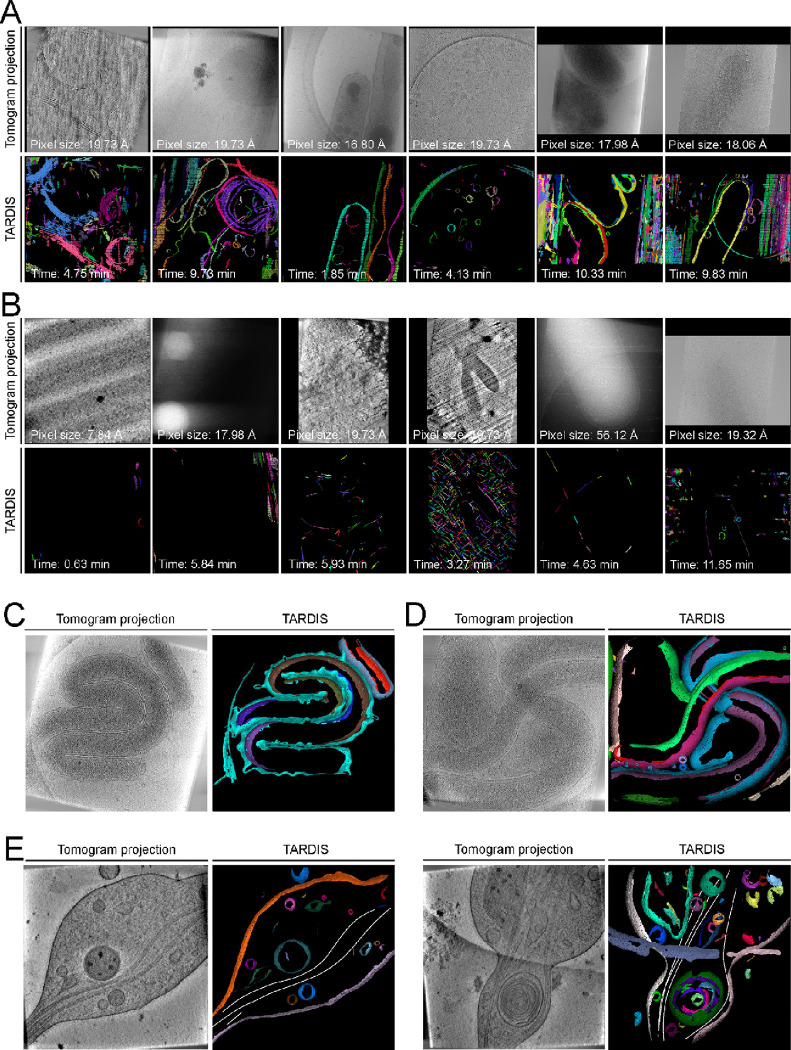
Gallery of example datasets predicted with TARDIS **A)** Examples of TARDIS predictions on randomly selected datasets from the CZI Cryo-ET data portal, with indicated pixel sizes and segmentation times. Colors represent individual instances. **B)** Example of a TARDIS prediction on a randomly selected dataset identified by TARDIS as containing no membrane structures. **C)** Example of tomographic slice and 3D view of segmented membraned from *Hylemonella gracilis*. **D)** Example of tomographic slice and 3D view of segmented membraned from *Hylemonella gracilis* in a presence of *Bdellovibrio*. **E)** Example of TARDIS segmentation of microtubules and membrane from EMPIAR-10815 dataset.

To demonstrate this, we segmented over 13,000 tomograms from the CZI Cryo-ET data portal enabling comparative biophysical analyses of membranes across various cell types (Fig. 5A–B), with pixel resolutions ranging from 0.867 to 56.12 Å. TARDIS autonomously processed these datasets in an average of 3.75 ±3.38 (mean ± standard deviation) minutes per tomogram, reducing the total segmentation time from an estimated 35 years (assuming one day per tomogram) to just one month.

This efficiency opens new avenues for analyzing cellular architecture. For example, automated segmentation revealed that 8.51% of tomograms lacked membrane structures or were of poor quality (Fig. 5A–B). The remaining tomograms contained an average of 5.59 ±7.26 (mean ± standard deviation) membranes per tomogram, with volumes of 0.009 ±0.017 μm³ (mean ± standard deviation). These large-scale quantitative insights, previously unattainable, now enable deeper exploration of cellular morphology and organization.

To further demonstrate TARDIS capabilities, we segmented 75 tomograms from the DS-10160 and DS-10161 CZI datasets^18^ of the bacterium *Hylemonella gracilis* under varying conditions, completing the task in ~5 hours on a single A100 GPU. This analysis revealed inner and outer membrane curvatures of 0.026 and 0.019, respectively. Exposure to *Bdellovibrio* shifted these values to 0.023 and 0.021, suggesting membrane softening or remodeling in response to the predator^46^ (Fig. 5C–D).

Additionally, we applied TARDIS to 30 tomograms from the EMPIAR-10815 dataset^47^, focusing on axons from mouse dorsal root ganglia. In ~2 hours, we segmented both membranes and microtubules, identifying an average of 4.8 ±2.5 (mean ± standard deviation) microtubules per field of view (Fig. 5E). Notably, 3 microtubules per field were consistently located within 50 nm of a membrane, with an average length of 173 nm, indicating potential microtubule-membrane interactions that may support anchoring and transport functions.

In conclusion, TARDIS streamlines segmentation and enhances comparative biophysical analyses, enabling researchers to investigate structural variations across diverse biological contexts. These analyses provide deeper insights into cellular adaptations, broadening our understanding of structure-function relationships across biological systems.

## Discussion

Structural biology has advanced significantly with innovations in imaging techniques and computational tools. However, accurately and efficiently segmenting tomograms remains a challenge due to the intricate and variable nature of biological structures. Traditional tools, such as Amira and MemBrain-seg V2, have been instrumental but often lack the precision and adaptability needed for diverse data types and varying quality levels.

A key feature of TARDIS is its robust instance segmentation capability, essential for analyzing complex biological structures where identical or similar macromolecules coexist in close proximity. TARDIS demonstrates state-of-the-art performance in instance segmentation tasks for both microtubules and membranes. Furthermore, its unique generalization capability allows TARDIS to extend to other instance segmentation problems without requiring retraining or fine-tuning. This flexibility positions TARDIS as a versatile tool for researchers working with diverse and complex cellular architectures. Its adaptability also facilitates rapid integration of new workflows for additional macromolecules, ensuring continued relevance in evolving research contexts.

Central to TARDIS is the incorporation of FNet, a specialized convolutional neural network architecture that enhances the accuracy of the semantic segmentation. Our results demonstrate that TARDIS, leveraging the FNet architecture, achieves higher precision in semantic segmentation compared to other methods. Specifically, the TARDIS FNet outperforms the standard UNet by producing sharper probability masks and capturing finer structural details (Suppl. Fig. 9A–C). Our DIST model further refines macromolecule segmentations by identifying individual object instances within the semantic masks output by FNet, effectively addressing issues of over-segmentation and under-segmentation commonly observed in other tools. DIST demonstrates a remarkable ability to generalize across different filamentous/linear and surface-like structures allowing it to be integrated seamlessly with new semantic segmentation methods. We show this by applying our pre-trained DIST model to various cellular macromolecules. Additionally, DIST can be extended to other point cloud data, including those derived from LiDAR scans, showcasing its broad applicability (Suppl. Results and Suppl. Fig. 9D).

A major advantage of TARDIS is its fully automated approach. It operates effectively across various dataset modalities, resolutions, and quality levels without requiring hyperparameter tuning or model retraining. This versatility streamlines the segmentation process and minimizes the need for manual intervention, making TARDIS an invaluable tool for structural biologists. To further enhance user interaction, our Napari plugin enables real-time observation of segmentation results and facilitates a more intuitive user experience.

TARDIS is quickly being adopted by the community and early versions of TARDIS have already been applied successfully to multiple biological studies, confirming its practical utility^26,48^. These early adopters have demonstrated TARDIS’s ability to deliver high-quality segmentations across diverse and complex biological structures, establishing it as a key resource in the field.

TARDIS represents a significant advancement in the field of structural biology by offering a highly precise, flexible, and automated solution for tomogram segmentation. The FNet architecture and integrated DIST model enable superior performance in both semantic and instance segmentation tasks, allowing pre-trained networks in TARDIS to outperform existing tools like UNet and MemBrain-Seg V2. The ability to operate across diverse datasets without the need for retraining and user-friendly tools such as the Napari plugin make TARDIS accessible and easy to use.

By making our datasets and code publicly available, we hope to encourage an open science environment that will drive further innovations and discoveries in understanding the complex architecture of cellular macromolecules. By incorporating additional segmentation networks for new macromolecules over time, we anticipate that TARDIS will grow into a comprehensive framework capable of annotating all cellular structures. We expect TARDIS will become an integral part of the study of large biomolecules.

## Methods

### Datasets

#### Microtubule tomographic and TIRF datasets

To develop microtubule dataset, we assembled a comprehensive collection of tomograms obtained under both room and cryogenic temperatures (Supp. Table 1). This dataset includes 93 tomograms with resolutions ranging from 1.16 to 3.64 nm. We divided the dataset into training, testing, and validation subsets using a 70/20/10 split. The collection encompasses three different imaging techniques, and seven specimen preservation methods and includes samples from eight distinct species, enhancing its diversity.

Microtubule segmentation was initially performed automatically using the AmiraZIBEdition software^39^, and subsequently refined manually according to established protocols^49^. The segmentation results were saved in Amira proprietary ASCII format, detailing each microtubule instance as a set of points. For semantic segmentation, we created binary masks from point sets by drawing splines along each instance with a diameter of 25 nm^50^. This approach effectively transforms the detailed instance data into a format suitable for training and evaluating our semantic segmentation model.

We also collected a dataset from interference reflection microscopy (IRM)/TIRF microscopy. The dataset includes TIRF images and movies acquired with Nikon Ti-E microscope equipped with an H-TIRF System (Nikon, Japan), a 60× oil immersion 1.49 NA TIRF objective and an Andor iXon Ultra EMCCD camera (Andor Technology, Belfast, UK). Double-stabilized porcine microtubules were visualized using the IRM channel, while Janelia 549 labeled microtubule-binding proteins were visualized with a 561 nm excitation laser. Segmentation was performed manually in Fiji and the annotations were converted to comma-separated values (CSV) file format.

#### Membrane tomographic dataset

For our membrane dataset, we face a similar challenge to the microtubule scenario, a lack of existing datasets for tomographic reconstructions or micrographs. To address this, we assemble a diverse dataset of membranes based on expert curation. We also included tomograms featuring membrane structures from the EMPIAR and CZI Cryo-ET data portals to further broaden the dataset.

This effort results in a dataset of 53 tomograms with resolutions ranging from 0.55 to 2.40 nm. We divide the dataset into training, testing, and validation segments with a 70/20/10 split. Segmentation was carried out in two stages. First, we hand-segment 10 tomograms to train our initial FNet model, after which we use this pre-trained model to perform all following segmentation. We employed Napari with a custom interpolation plugin to curate all membranes segmentation.

Using our segmentations, we extract instances from binary masks through a connected component algorithm. Point clouds were generated via skeletonization and voxelization, ensuring a consistent and accurate representation of membrane structures in our dataset.

#### Membrane micrograph dataset

In addition to assembling a membrane dataset from tomographic reconstructions, we enhance our training and validation resources for CNN models by incorporating micrographs. This expansion involves not only using micrographs provided by collaborators but also simulating micrographs through tomogram projections. We achieve this by selecting tomographic volumes approximately 60 nm thick and applying z-max projections to replicate the appearance of micrographs. This approach significantly diversifies our dataset, allowing us to include a broader range of data types. The methods for semantic and instance segmentation of these simulated micrographs adhere to the same procedures previously outlined.

#### Actin tomographic dataset

We assemble tomograms of actin obtained under cryogenic temperatures (Supp. Table 1). In total we fully annotated actin in 3 tomograms with resolution 1.38 nm and 1.81 nm. We divide the dataset into training, testing, and validation segments with a 70/20/10 split.

#### Point Cloud synthetic dataset

To train the DIST model, we construct a dataset combining actual and simulated data. Real images of membranes and microtubules were hand-segmented to create ground truth masks, which are then skeletonized and voxelized. Simulated point clouds are also generated for filaments and membranes, and processed using the same methods as the real data. This approach ensures consistency between simulated and real datasets. To further augment simulated dataset, each generated point is jiggled in random direction and amplitude to imitate image noise. Additionally, simulated datasets were further augmented with random noise set-up to 10% of the number of points and random drop of points within instances of microtubules and membranes. Each point cloud is annotated with the correct instance IDs based on known ground truth positions. The dataset is divided into 80% for training and 20% for validation, with the simulated data included solely in the training set, comprising 50% of the total training dataset.

### Data preprocessing for CNN

Data preprocessing and augmentation are crucial for optimizing model performance. Initially, each micrograph and tomogram underwent intensity normalization, where the mean intensity is subtracted and divided by the standard deviation, followed by scaling all values to a range between −1 and 1. To manage large images, we cut them into overlapping patches - 256×256 pixels for micrographs and 96x96x96 voxels for tomograms. The datasets are then divided into training, testing, and validation (Supplementary Table 1).

### FNet CNN model for accurate semantic segmentation

The TARDIS semantic segmentation workflow relies on the FNet model, illustrated in Supplementary Figure 1, which features an encoder-decoder architecture with dual decoder branches, giving the network an F-like shape. The first decoder adheres to a traditional UNet design, employing skip connections to maintain spatial detail, while the secondary decoder incorporates hierarchical skip connections from all encoder levels, enhancing the model ability to capture features at multiple scales. This dual-decoder setup is finalized with a single convolution and sigmoid function for binary segmentation. Both FNet and UNet architectures include five layers each for encoding and decoding. Each layer is composed of group normalization, a dual-convolution block followed by a LeakyReLu^51^ activation function. The training was conducted using the NAdam optimizer with a learning rate of 0.0001 and binary cross-entropy (BCE) as the loss function. To standardize training between different modules and avoid overfitting, an early stopping criterion was applied, halting training if no validation loss improvement was observed over 250 epochs.

### Post-processing of semantic binary masks to point clouds

The initial segmentation produces binary masks for filaments and membranes, which include both false-positives and false-negatives (Supp. Fig. 2A). These inaccuracies affect the precision of subsequent instance segmentation tasks, such as those using connected component algorithms. To improve this, we convert the binary masks into point clouds through a series of steps.

First, we perform skeletonization of the binary masks to capture the core structure, followed by voxel down-sampling. The skeletonization process starts with a Euclidean distance transformation, which computes the shortest distance from each foreground pixel to the nearest background pixel, creating a distance map of the mask. We then apply thresholding based on specific feature sizes - 12.5 nm for microtubules and 2 nm for membranes - to reduce noise and separate closely packed structures (Supp. Fig. 2A).

Next, we generate point clouds by recording the coordinates of each pixel in the skeleton. Given that these point clouds can become very dense, sometimes exceeding one million points, we use voxel down-sampling to reduce the point count by a factor of ten without losing spatial and geometric detail. This down-sampling yields a simplified yet accurate representation of the original structures (Supp. Fig. 2B), enhancing computational efficiency and manageability.

### DIST model for point cloud instance segmentation

Instance segmentation with DIST is divided into three parts: 1) Transformation of the raw point cloud into a pairwise representation based on the distances between points to capture the initial edge features. 2) Processing through the DIST model to output a matrix representing the probability of connection between each point pair, as shown in Supplementary Figure 3C. This matrix predicts potential edges in the instance graph, forming the basis for distinguishing individual instances. 3) Application of a graph cut algorithm on the maximum likelihood graph to segment instances based on the predicted edges.

Training of the DIST model focuses on predicting these edges by learning vector representations for each point pair within the cloud, termed pairwise or edge embeddings. To achieve translation and rotation invariance, initial edge features are based on the Euclidean distances between point pairs, employing a scaled exponential function of the negative squared distance to prioritize proximity. Formally, for a point cloud comprising points $p_{i}(i\;=\;1,\;.\;.\;.\;,\;n)$, we construct a graph G with nodes for each $p_{i}$ and edges $e_{i,j}$ connecting all pairs, where the weight $e_{i,j}$ is calculated as $exp\left(-{d^{2}}_{i,j}/\left(2\times s^{2}\right)\right)$, with $d_{i,j}$ denoting the Euclidean distance between $p_{i}$ and $p_{j}$, and s representing a predetermined scale factor. This weighting approach allows us to embed geometrical information about all points directly from the Euclidean distance. This allowed us to ensure the SO(n) invariance for translation and rotation and embed all geometrical information about nodes geometrical relationship and position in the space. This approach effectively addresses the challenges posed by the complex nature of objects in point clouds, especially in bioimaging domains where these objects can be large and may intersect or overlap.

### Training DIST model for instance segmentation

We train two general DIST models (Supp. Fig 3B). The first segments chain-like structures, for example microtubule filaments or 2D membranes derived from cryo-EM micrographs. The second segments 3D surfaces, like membranes, mitochondria, etc. Similarly, as described before DIST was trained using the NAdam optimizer with a 0.00001 learning rate. An early stop mechanism was employed to halt training when there was no improvement in the moving average of validation loss over 100 consecutive epochs. Regarding the loss function, binary cross-entropy (BCE) was used to train the DIST model.

Training and evaluating the DIST model required building an instance graph representation for each point cloud. The graph is represented as a 2D matrix (Supp. Fig 3A) with nodes p represented by coordinates in n-dimensional space, and the edge represents spatial connectivity between two nodes $p_{i}$ and $p_{j}$, where i is shown in the matrix row, and j in the matrix column. The graph representation for linear-like structures (membranes in 2D and microtubules) is constructed from ground truth annotations in which each instance is an ordered list of nodes. Knowing the order, the edge matrix is defined as 1 for each neighboring $p_{i,j}$. This approach imposes restrictions where each i can have only up to two edges. In the case of 3D structures like membranes, we build this 2D matrix by establishing for each $p_{i}$ edge connection to its eight nearest neighbor $p_{j}$ within the same instance.

### Graph cut from the predicted adjacency matrix

The output of the DIST model is a probability adjacency matrix (Supp. Fig. 3A), which contains the probability of an edge existing between a set of two points. For the segmentation of filament and membrane structures, which are known to have a linear chain we know the prior that each node (points) has at most two neighbors. We can use this information to search for the graph chain of nodes, where each node is connected to two other nodes with the highest probability. In this case, we adopt a greedy algorithm for graph inference. First, we build a hash map for each node in the point cloud containing node ID, and edge probability $p_{i,j}$. Next, for each new instance, we searched the hash map for the initial node. This allows us in the final step to iteratively find up to two edges with the highest probability for all connected nodes. This process is continued until no new edges can be recognized for a given instance. We use this approach when segmenting membranes and microtubules that follow this chain structure. In the case of 3D structures like membrane volumes, we do not have such priors, therefore we use a greedy graph cut algorithm that connects nodes with all other nodes if their probability of connection is higher than 0.5. This process continues until no new edges can be identified for a given instance. The output of this approach is a list of coordinates belonging to the same instance which can be output in any format suitable for the user.

### Benchmark experiments

#### Semantic Segmentation.

In the evaluation of the semantic segmentation capabilities of the TARDIS framework, we employed a comprehensive set of metrics, including F1 score, precision, and recall (Eq.1–3), alongside more nuanced measures such as average precision (AP; Eq. 4) and average precision at a recall threshold of 90% (AP90; Eq. 4). These metrics collectively offer a robust assessment of the segmentation accuracy, effectively capturing the performance of TARDIS in prioritizing true positives over false positives and negatives. The AP and AP90 scores provide insights into the model efficacy across varying thresholds of detection confidence, reflecting the area under the precision-recall curve and thus offering a detailed picture of model performance in semantic segmentation tasks.

#### Instance Segmentation

For the instance segmentation evaluation, TARDIS was assessed using the mean coverage (mCov; Eq. 5) metric. The mCov metric is designed to accurately quantify the overlap between the ground-truth instances and the predicted instances within the point cloud, using intersection over union (IoU) as the basis of measurement. This approach ensures an accurate evaluation of instance segmentation.

#### Naïve Distance Grouping

In the absence of a pre-existing baseline benchmark for evaluating the performance of similar point cloud data derived from filaments and membranes, we devised our benchmarking standard, rooted in the principle of naive distance grouping. This approach entailed computing the average distance between neighboring points within each point cloud dataset. This average distance served as a critical distance threshold for each node (point) in the dataset. To delineate instances within the data, we grouped points based on the premise that any given node could be connected to a maximum of either two other nodes or an indefinite number (infinity) of nodes, provided these connections did not exceed the established threshold distance. This method allowed us to programmatically retrieve instances from complex point cloud datasets by leveraging spatial proximity.

## Supplementary Material

Supplement 1

## Figures and Tables

**Table 1:** Semantic segmentation metrics for microtubule filaments segmented from tomographic dataset. Precision, recall, and F1 scores are calculated using a probability threshold of 0.5 for TARDIS and Amira.

	Model	F1	Precision	Recall	AP	AP90
All Datasets	**TARDIS**	**0.61**	**0.70**	**0.55**	**0.69**	0.33
	Amira	0.17	0.11	0.51	0.25	0.01
Cryo-ET dataset	**TARDIS**	**0.58**	**0.72**	0.48	**0.71**	0.20
	Amira	0.03	0.04	0.06	0.02	0.02
TEM dataset	**TARDIS**	0.61	0.70	**0.56**	**0.67**	0.35
	Amira	0.20	0.12	0.61	0.48	0.01

**Table 2:** Semantic segmentation metrics for membranes se gmented from cryo-ET

Type	F1	Precision	Recall	AP	AP90
**TARDIS**	**0.66**	0.68	0.65	0.58	**0.31**
MemBrain-seg V2	0.40	0.27	**0.72**	0.24	0.18
TomoSegMemTV	0.21	0.17	0.42	-	-

**Table 3:** Semantic segmentation metrics for membranes se 802 gmented from cryo-ET

	Type	F1	Precision	Recall	AP	AP90
All dataset	**TARDIS**	**0.56**	0.62	**0.55**	**0.61**	**0.37**
	UNet	0.54	**0.64**	0.51	0.61	0.37
Dataset with high-resolution* membrane	**TARDIS**	**0.64**	0.73	**0.62**	**0.71**	**0.52**
	UNet	0.62	**0.74**	0.58	0.71	0.52
Dataset with low-resolution** membrane	**TARDIS**	**0.41**	0.44	**0.42**	0.44	**0.12**
	UNet	0.40	**0.47**	0.37	**0.45**	0.10

* high-resolution - Micrograph of resolution smaller or equal to 10 Å

** low-resolution - Micrograph of resolution greater than 10 Å
