# Supplementary material for: Accurate and fast segmentation of filaments and membranes in micrographs and tomograms with TARDIS
Source: bioRxiv. 2024 Dec 20:2024.12.19.629196. Preprint. [Version 1] doi: 10.1101/2024.12.19.629196 (PMC11702698; doi:10.1101/2024.12.19.629196)
Supplement: Supplementary file 1 [file NIHPP2024.12.19.629196v1-supplement-1.pdf]

bioRxiv preprint doi: https://doi.org/10.1101/2024.12.19.629196; this version posted December 20, 2024. The copyright holder for this preprint (which was not certified by peer review) is the author/funder, who has granted bioRxiv a license to display the preprint in perpetuity. It is made available under aCC-BY 4.0 International license.

Supplement

Ablation study

TARDIS improves semantic segmentation performance due to the FNet architecture

TARDIS incorporates a novel CNN architecture called FNet into its workflow. We evaluated FNet on semantic segmentation tasks involving microtubules and membranes and found that the TARDIS FNet model significantly outperforms current state-of-the-art tools (**Table 1-3**). To compare TARDIS FNet efficiency also against other CNN architecture, we trained both FNet and a similarly deep UNet model within TARDIS, ensuring identical training parameters for a fair comparison. As expected, the TARDIS FNet model achieved superior results compared to the TARDIS UNet model across both microtubule and membrane benchmarks (**Suppl. Fig. 7A-C**). Specifically, the FNet model attained better metrics for microtubule segmentation from both Cryo-ET and plastic ET datasets (**Suppl. Fig. 7A, Supp. Fig 8A** and **Suppl. Table 5**). Additionally, TARDIS FNet demonstrated significant performance improvements in membrane segmentation tasks using data from both Cryo-ET (**Suppl. Fig. 7B, Supp. Fig 8B** and **Suppl. Table 6**) and micrographs datasets (**Suppl. Fig. 7C, Supp. Fig 8C** and **Suppl. Table 7**). Overall, the TARDIS FNet model demonstrated greater performance compared to a similarly deep UNet model. This improvement was achieved through our dual-decoder strategy, which enabled the generation of sharper probability maps (**Suppl. Fig 9**)

To further increase the reliability of TARDIS semantic segmentation, we implemented a pixel size normalization strategy. This approach ensures that various objects segmented by our TARDIS semantic segmentation module maintain at a consistent scale. We determined the optimal pixel size scaling for membrane segmentation tasks in both tomograms and micrographs by training the TARDIS FNet and UNet models with pixel normalization set to 15 Å,

bioRxiv preprint doi: https://doi.org/10.1101/2024.12.19.629196; this version posted December 20, 2024. The copyright holder for this preprint (which was not certified by peer review) is the author/funder, who has granted bioRxiv a license to display the preprint in perpetuity. It is made available under aCC-BY 4.0 International license.

8 Å, and 4 Å using micrograph datasets. Our results indicated that for tomographic datasets, the TARDIS FNet model performed best when tomograms were scaled to a resolution of 15Å (**Suppl. Fig. 8D** and **Suppl. Table 6**). In the case of micrographs, optimal performance was achieved when the models were trained on data normalized to a resolution of 4Å (**Suppl. Fig. 8C** and **Suppl. Table 7**). Additionally, we observed that the TARDIS FNet model set to 4Å yielded the best results particularly for high-resolution micrographs (<10 Å), while for low-resolution micrographs, scaling to 8 Å provided the most accurate segmentation. Based on these findings, we have integrated the optimal pixel size normalization into our automated workflow and publicly released the enhanced model.

DIST can segment other point cloud data

DIST was initially developed for cryo-electron microscopy data, but it can be also effectively applied to point cloud segmentation in domains like LiDAR from the ScanNetV2 dataset or the PartNet dataset. To test this, we applied the DIST model, specifically pre-trained on the LiDAR dataset, to segment point clouds from the ScanNetV2 and PartNet datasets. Our results demonstrate that DIST performs well on this task, effectively segmenting objects and parts (**Suppl. Fig 9D**). This shows that the DIST model generalizes well across datasets and adapt for diverse point cloud segmentation challenges, further validating its potential for tasks beyond its original bioimaging application.

bioRxiv preprint doi: https://doi.org/10.1101/2024.12.19.629196; this version posted December 20, 2024. The copyright holder for this preprint (which was not certified by peer review) is the author/funder, who has granted bioRxiv a license to display the preprint in perpetuity. It is made available under aCC-BY 4.0 International license.

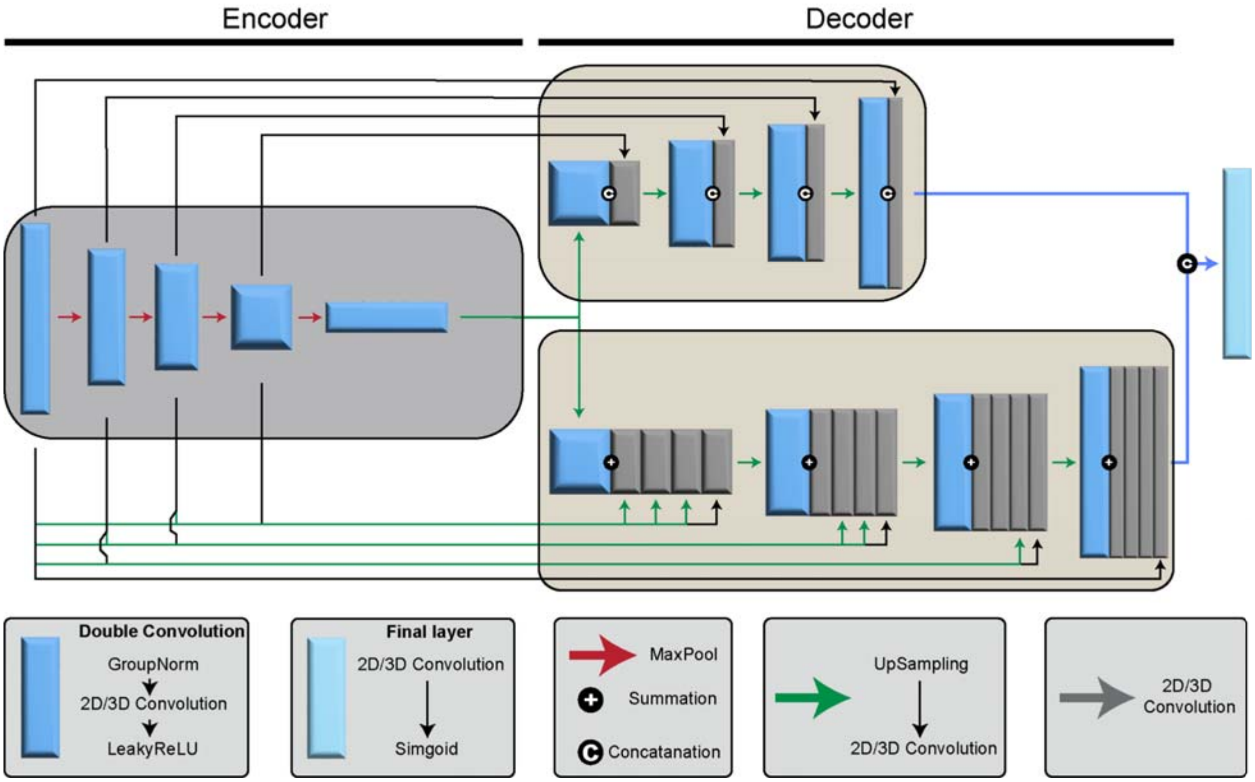

852

853 **Supplementary Figure 1: FNet CNN model architecture**

854 The figure illustrates a CNN encoder-decoder framework dedicated to semantic segmentation in
855 images with low signal-to-noise ratios.

bioRxiv preprint doi: https://doi.org/10.1101/2024.12.19.629196; this version posted December 20, 2024. The copyright holder for this preprint (which was not certified by peer review) is the author/funder, who has granted bioRxiv a license to display the preprint in perpetuity. It is made available under aCC-BY 4.0 International license.

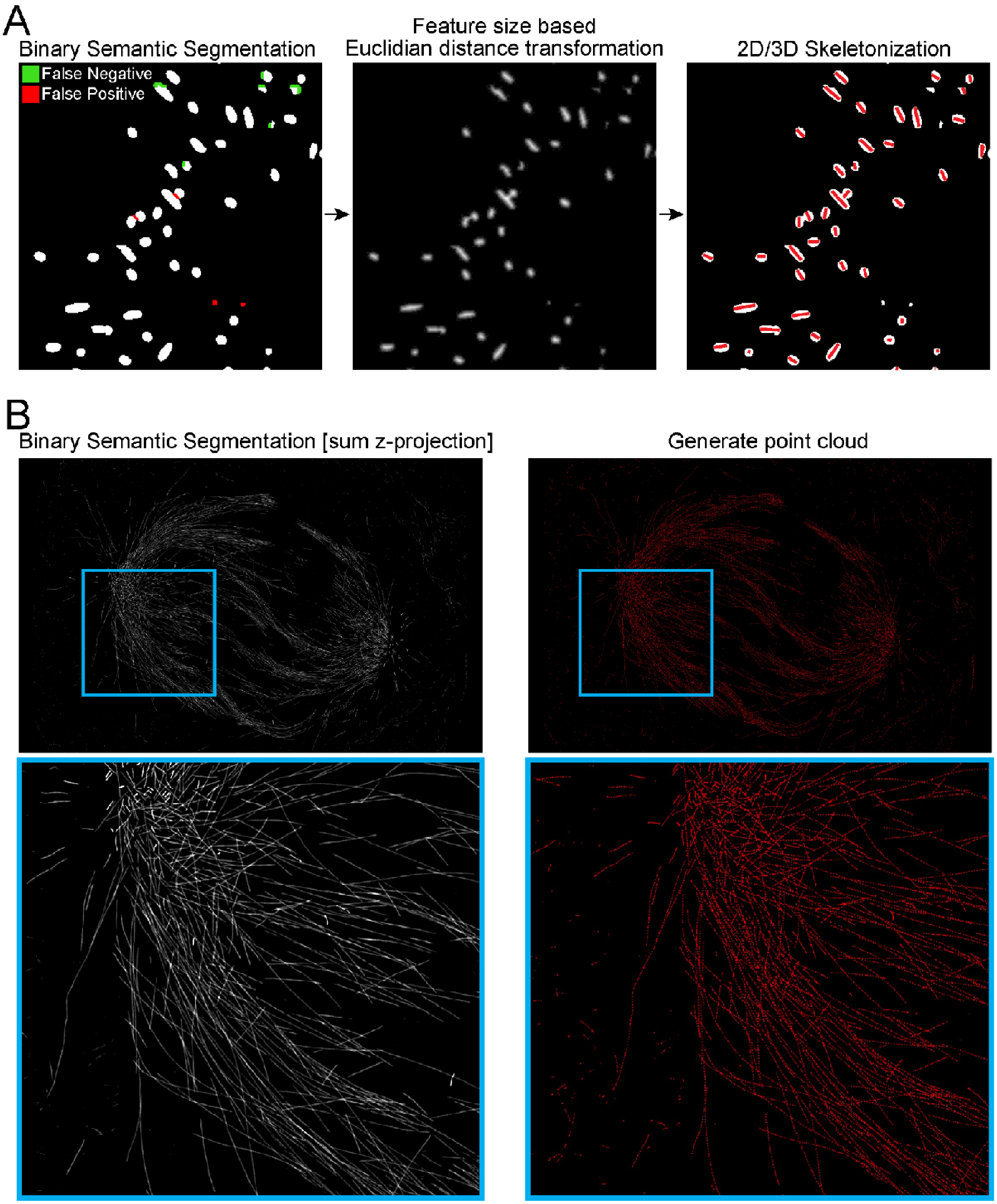

856

857 **Supplementary Figure 2: Illustration of pre-processing steps after the semantic**
858 **segmentation**

bioRxiv preprint doi: https://doi.org/10.1101/2024.12.19.629196; this version posted December 20, 2024. The copyright holder for this preprint (which was not certified by peer review) is the author/funder, who has granted bioRxiv a license to display the preprint in perpetuity. It is made available under aCC-BY 4.0 International license.

A) Illustration of converting binary semantic masks to a point cloud. Green highlights false negatives and red indicates false positives. The process begins with applying Euclidean distance transformation based on feature size to minimize segmentation errors. This is followed by skeletonization to extract the core features of the mask, which are then represented as a point cloud. **B)** Example of TARDIS binary semantic segmentation mask and the corresponding point cloud. The gray color represents a z-sum projection of the semantic mask, while the red color denotes individual points within the generated point cloud.

bioRxiv preprint doi: https://doi.org/10.1101/2024.12.19.629196; this version posted December 20, 2024. The copyright holder for this preprint (which was not certified by peer review) is the author/funder, who has granted bioRxiv a license to display the preprint in perpetuity. It is made available under aCC-BY 4.0 International license.

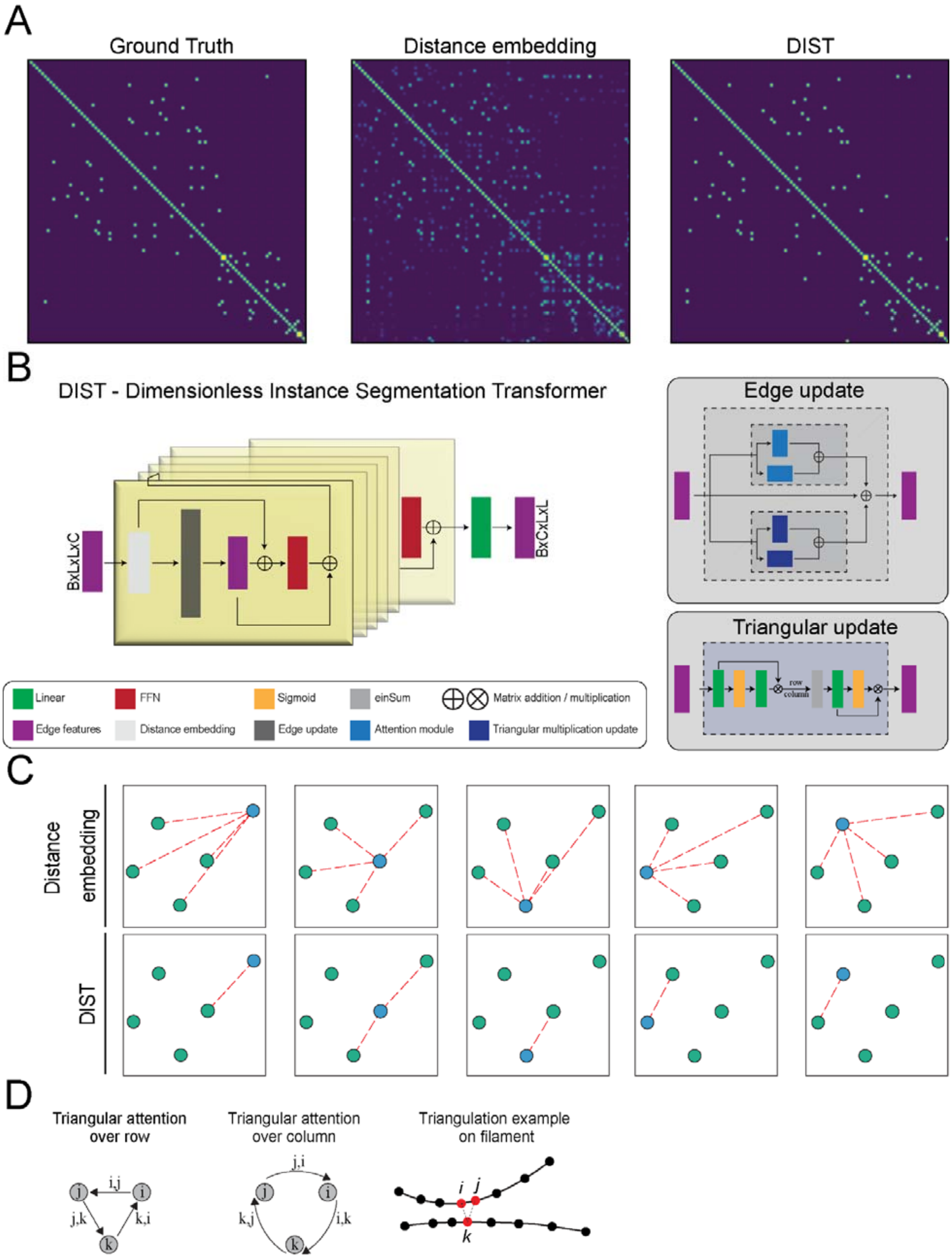

866

bioRxiv preprint doi: https://doi.org/10.1101/2024.12.19.629196; this version posted December 20, 2024. The copyright holder for this preprint (which was not certified by peer review) is the author/funder, who has granted bioRxiv a license to display the preprint in perpetuity. It is made available under aCC-BY 4.0 International license.

Supplementary Figure 3: The DIST model architecture

A) Adjacency graphs illustrating edge connectivity in point clouds. The first graph displays the ground truth adjacency matrix with each node connected to its two nearest neighbors. The second graph shows the adjacency matrix derived from node distances and predicted probabilities using DIST. **B)** Detailed workflow of DIST starts with edge representations initialization based on distances between points in the input point cloud, resulting in an SO(n) invariant representation. DIST layers then refine edge features through triangular multiplicative updates and axial attention modules[52], ultimately decoding these into edge probabilities. Arrows indicate the flow of information throughout the process. **C)** A cartoon depicting the distinction between initial distance embeddings and DIST predictions. Initially, each node connects to all other nodes, whereas DIST predictions retain only the relevant edges. **D)** Illustration of a triangular multiplicative update. Pairs of nodes are connected as edges on the graph, with the update process demonstrated by a triangle multiplicative update. Circles represent nodes, and arrows indicate the edges involved in the update.

bioRxiv preprint doi: https://doi.org/10.1101/2024.12.19.629196; this version posted December 20, 2024. The copyright holder for this preprint (which was not certified by peer review) is the author/funder, who has granted bioRxiv a license to display the preprint in perpetuity. It is made available under aCC-BY 4.0 International license.

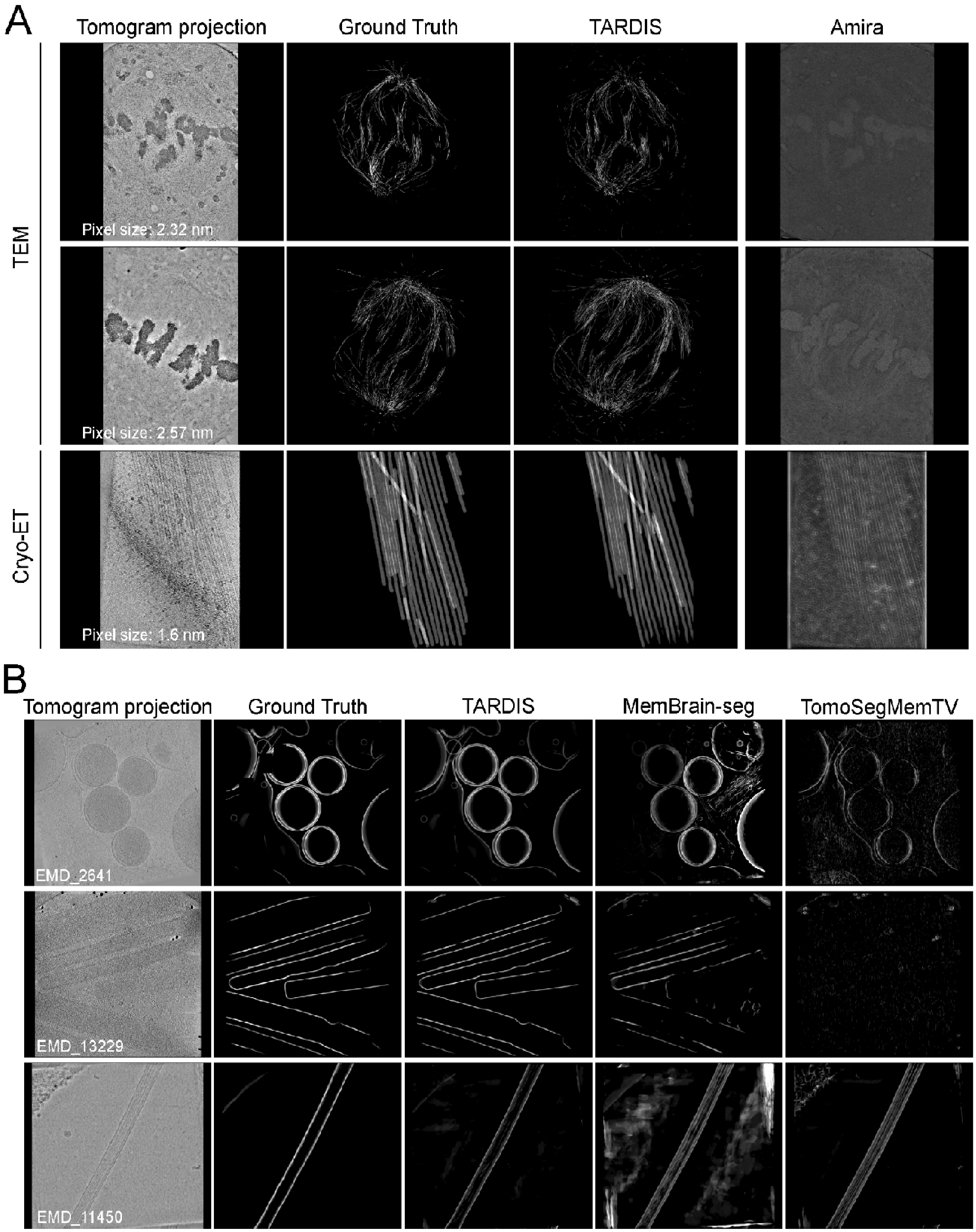

881

bioRxiv preprint doi: https://doi.org/10.1101/2024.12.19.629196; this version posted December 20, 2024. The copyright holder for this preprint (which was not certified by peer review) is the author/funder, who has granted bioRxiv a license to display the preprint in perpetuity. It is made available under aCC-BY 4.0 International license.

Supplementary Figure 4: Comparison of semantic segmentations using pre-trained FNet and UNet

A) Predicted semantic segmentation results for tomograms containing microtubules. **B)** Predicted semantic segmentation results for tomograms containing membranes, with the gray color representing a sum z-projection of the semantic mask.

bioRxiv preprint doi: https://doi.org/10.1101/2024.12.19.629196; this version posted December 20, 2024. The copyright holder for this preprint (which was not certified by peer review) is the author/funder, who has granted bioRxiv a license to display the preprint in perpetuity. It is made available under aCC-BY 4.0 International license.

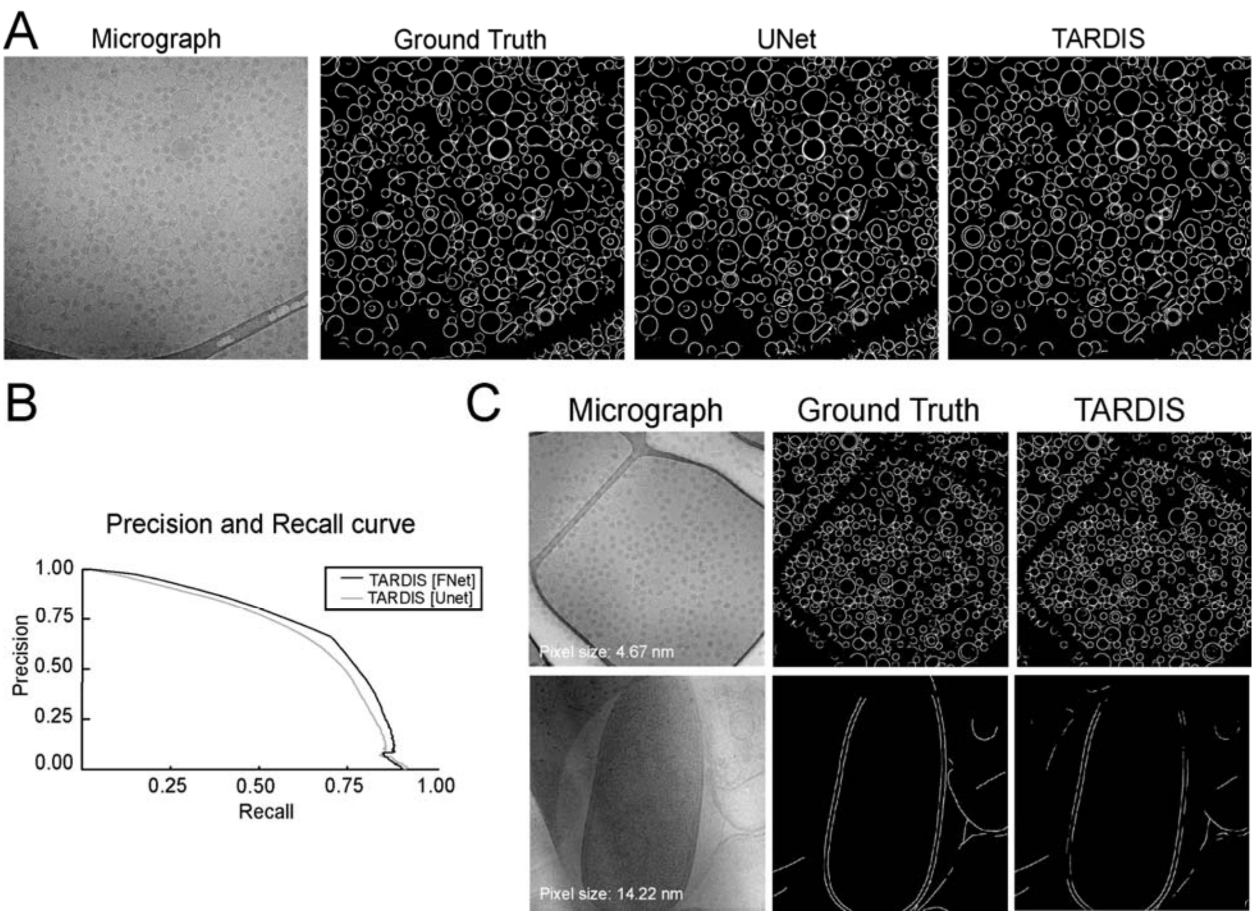

Supplementary Figure 5: Comparison of semantic segmentations of membranes from EM micrographs

A) Predicted semantic membrane segmentation results for micrographs containing enveloped virus and liposome particles using TARDIS FNet and UNet pre-trained models. **B)** Precision/Recall curves for microtubule segmentation benchmark datasets, comparing predictions made by Amira, the pre-trained UNet, and TARDIS. **C)** Predicted semantic segmentation results for micrographs containing virus and liposomal membranes, on high- and low-resolution.

bioRxiv preprint doi: https://doi.org/10.1101/2024.12.19.629196; this version posted December 20, 2024. The copyright holder for this preprint (which was not certified by peer review) is the author/funder, who has granted bioRxiv a license to display the preprint in perpetuity. It is made available under aCC-BY 4.0 International license.

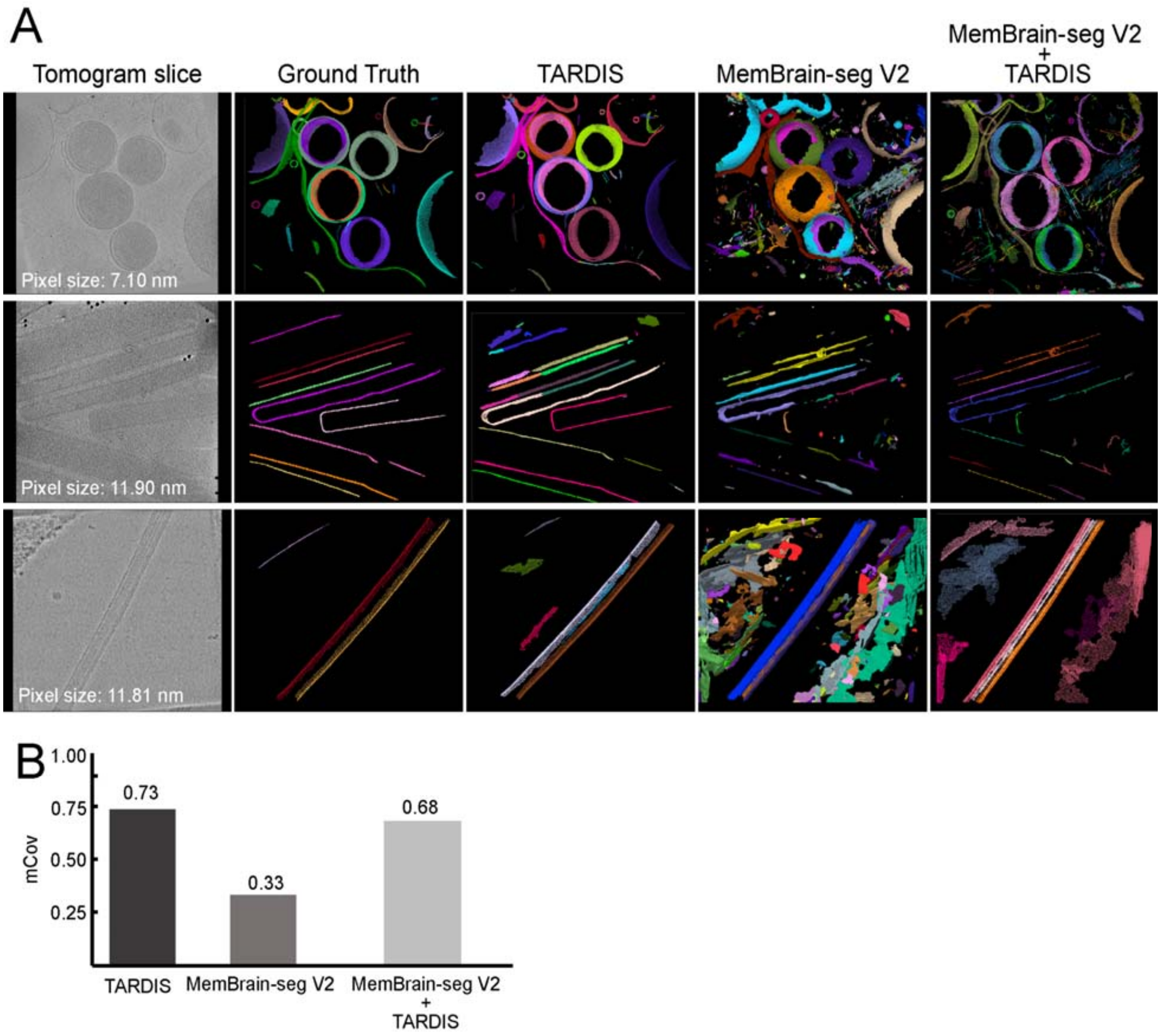

896

897 **Supplementary Figure 6: Comparison of results combining TARDIS workflow MemBrain-**
898 **seg V2**

899 **A)** Sum z-projections of predicted instance segmentations from each tomogram, with randomly
900 assigned colors representing individual instances. **B)** Plot displaying mCov scores for instance
901 segmentation predictions.

bioRxiv preprint doi: https://doi.org/10.1101/2024.12.19.629196; this version posted December 20, 2024. The copyright holder for this preprint (which was not certified by peer review) is the author/funder, who has granted bioRxiv a license to display the preprint in perpetuity. It is made available under aCC-BY 4.0 International license.

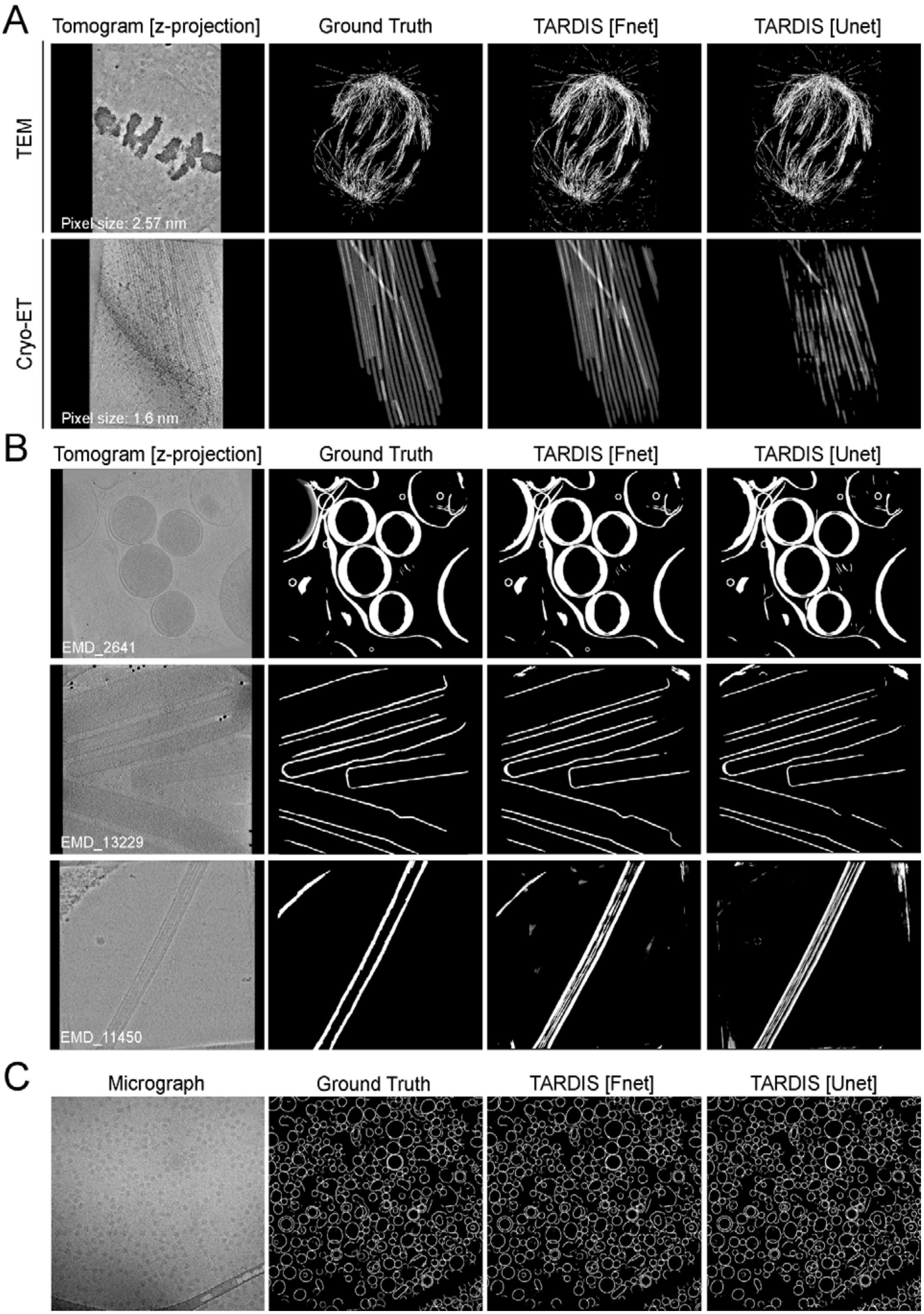

bioRxiv preprint doi: https://doi.org/10.1101/2024.12.19.629196; this version posted December 20, 2024. The copyright holder for this preprint (which was not certified by peer review) is the author/funder, who has granted bioRxiv a license to display the preprint in perpetuity. It is made available under aCC-BY 4.0 International license.

Supplementary Figure 7: Comparison of semantic segmentations between TARDIS FNet CNN model and UNet

A) Predicted semantic segmentation results for tomograms containing microtubules using TARDIS FNet and UNet pre-trained models. **B)** Predicted semantic segmentation results for tomograms containing membranes. **C)** Predicted semantic membrane segmentation results for micrographs containing enveloped virus and liposomes particles.

bioRxiv preprint doi: https://doi.org/10.1101/2024.12.19.629196; this version posted December 20, 2024. The copyright holder for this preprint (which was not certified by peer review) is the author/funder, who has granted bioRxiv a license to display the preprint in perpetuity. It is made available under aCC-BY 4.0 International license.

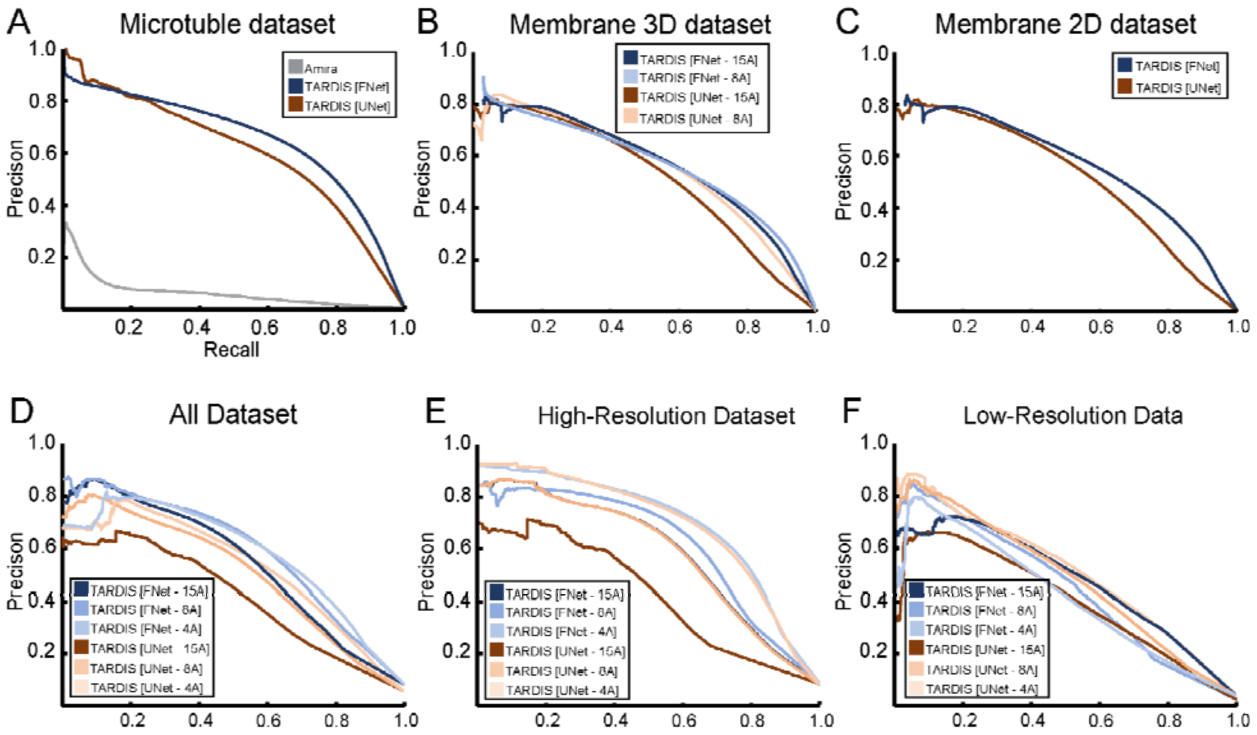

Supplementary Figure 8: Precision/Recall curves comparing semantic segmentation performance

A) Precision/Recall curves for microtubule segmentation benchmark datasets, comparing predictions made by Amira, the pre-trained UNet, and TARDIS. **B)** Precision/Recall curves for membrane segmentation benchmark datasets using the pre-trained FNet and UNet model on tomographic data. **C)** Precision/Recall curves for membrane segmentation benchmark datasets using TARDIS and the pre-trained UNet model on micrograph data. **D)** Precision and recall curves for membrane datasets benchmarked using the FNet and UNet model. Different lines represent UNet models trained with specific normalized pixel size resolutions. **E)** Precision and recall curves for high-resolution membrane dataset benchmarks using the FNet and UNet model. Different lines represent UNet models trained with various normalized pixel size resolutions. **F)** Precision and recall curves for low-resolution membrane dataset benchmarks using the FNet and UNet model. Different lines represent UNet models trained with various normalized pixel size resolutions.

bioRxiv preprint doi: https://doi.org/10.1101/2024.12.19.629196; this version posted December 20, 2024. The copyright holder for this preprint (which was not certified by peer review) is the author/funder, who has granted bioRxiv a license to display the preprint in perpetuity. It is made available under aCC-BY 4.0 International license.

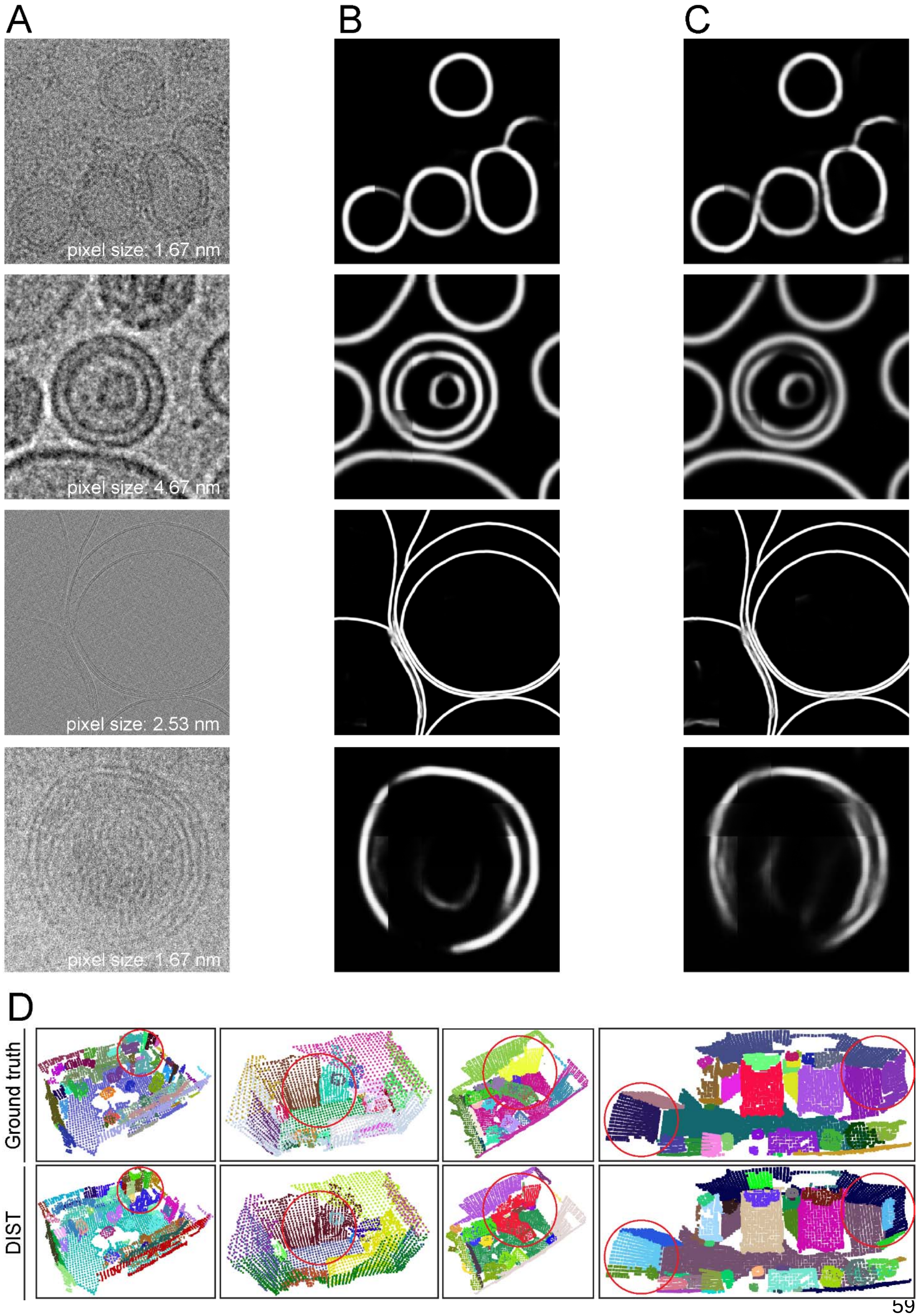

924

bioRxiv preprint doi: https://doi.org/10.1101/2024.12.19.629196; this version posted December 20, 2024. The copyright holder for this preprint (which was not certified by peer review) is the author/funder, who has granted bioRxiv a license to display the preprint in perpetuity. It is made available under aCC-BY 4.0 International license.

Supplementary Figure 9: Example of Unet and Fnet prediction on micrograph with membranes at various resolutions

A) Cropped micrographs showing selected areas containing membranes. **B)** Probability map generated by TARDIS semantic segmentation for the area depicted in A. **C)** Probability maps of the same areas shown in A, as predicted by the UNet model. **D)** Example of point clouds segmented with TARDIS using pre-trained model on LiDAR ScanNet V2 dataset. Read circles highlight areas of discrepancy between ground truth labels and DIST predictions.

bioRxiv preprint doi: https://doi.org/10.1101/2024.12.19.629196; this version posted December 20, 2024. The copyright holder for this preprint (which was not certified by peer review) is the author/funder, who has granted bioRxiv a license to display the preprint in perpetuity. It is made available under aCC-BY 4.0 International license.

Supplementary Table 1: List of datasets with indicated train/validation/evaluation split

Dataset	Origin	Sample acquired by	Imagin technique	Specimen	Content	Pixel size (ang/pix)	Total number of images	Number of objects	Publish
Actin	EMPIAR-10989	-	Cryo-ET	RPE-1 cells	actin filament	13.8	3	-	Yes
	J. Johnston	J. Johnston		-	actin filament	18.1	3	-	No
Microtubules - TIRF	Bařinka lab	L. Motlová	IRM/ TIRF	*In-vitro* microtubules	Double-stabilized porcine microtubules	750	112	1656	Yes
Microtubules – ET/Cryo-ET				HeLa Kyoto cells	Microtubules	23.2	27	17466	Yes
		R. Kiewisz	ET	U2OS cells	Microtubules	25.7	9	10223	Yes
	Müller-Reichert lab			RPE-1 cells	Microtubules	25.7	1	472	No
				C. elegans worm	Microtubules	23.2	1	441	Yes
		G. Fabig	ET	*C. elegans* worm	Microtubules	23.2 - 25.7	27	20223	No
				U2OS cells	Microtubules	25.7	5	4681	No
	Redemann lab	M. Magaj	ET	*C. elegans* worm	Microtubules	21.2	2	1199	Yes
		H. Yazdkhati	ET	*Drosophila* embryo	Microtubules	21.43	4	7001	Yes

bioRxiv preprint doi: https://doi.org/10.1101/2024.12.19.629196; this version posted December 20, 2024. The copyright holder for this preprint (which was not certified by peer review) is the author/funder, who has granted bioRxiv a license to display the preprint in perpetuity. It is made available under aCC-BY 4.0 International license.

		S. Redemann	ET	*C. elegans* worm	Microtubules	11.8 & 23.2	2	1784	Yes
	O. Clarke	W. Conway	Cryo-ET	*In-vitro* microtubules	Microtubules	36.27	2	189	Yes
	W. Conway	W. Conway	Cryo-ET	*In-vitro* microtubules	Microtubules	16	11	578	No
Membranes - 3D	CryoTomoSim	-	Simulated Cryo-ET	-	Membranes	13.3	2	11	Yes
	J. Johnston	J. Johnston	Cryo-ET	-	Membrane	13.6	4	80	No
	Shee-Mei lab	V. Kostyuchenko	Cryo-ET	-	Membrane/ Viruses	6.8 – 13.5	8	89	No
	O. Clarke	A. Nobel & J. Johnston	Cryo-ET	-	Membrane	8 – 16.56	6	172	Yes
	CZI Data-Portal	-	Cryo-ET	-	Membrane/Liposomes	6.4 – 24.7	16	337	Yes
	EMPIAR	-	Cryo-ET	-	Membrane/Liposomes	5.5 – 17.6	17	260	Yes
Membrane – 2D	T. Bepler	H. Hui	Cryo-EM	-	Liposomes	1.3	45	1187	Yes
	F. Heberle	F. Heberle	Cryo-EM	-	Liposomes	2.5	5	326	Yes
	Shee-Mei lab	V. Kostyuchenko	Cryo-EM	-	Membrane/ Viruses	4.7	5	2041	Yes
	-	-	Cryo-ET	Projected Cryo-ET tomograms	Membrane/ Viruses/Liposomes	1.7 – 16.6	65	1331	Yes

bioRxiv preprint doi: https://doi.org/10.1101/2024.12.19.629196; this version posted December 20, 2024. The copyright holder for this preprint (which was not certified by peer review) is the author/funder, who has granted bioRxiv a license to display the preprint in perpetuity. It is made available under aCC-BY 4.0 International license.

Supplementary Table 2: Validation of a point cloud segmentation on ground truth data using DIST

	Filament-like object	Surface structures
DIST (linear / surface)	**0.96**	**0.91**
Distance grouping	0.21	0.25

bioRxiv preprint doi: https://doi.org/10.1101/2024.12.19.629196; this version posted December 20, 2024. The copyright holder for this preprint (which was not certified by peer review) is the author/funder, who has granted bioRxiv a license to display the preprint in perpetuity. It is made available under aCC-BY 4.0 International license.

Supplementary Table 3: Validation of an influence of cryo-ET projection on improving semantic segmentation of Cryo-EM data

	Scaling	Type	F1	Precision	Recall	AP	AP90
All Dataset	**8A**	**TARDIS**	**0.51**	**0.71**	0.44	**0.68**	**0.37**
	8A [Only Cryo-EM]	TARDIS	0.46	0.61	0.47	0.56	0.25
Dataset with high-resolution membrane	8A	TARDIS	0.54	**0.76**	0.48	0.72	**0.48**
	**8A [Only Cryo-EM**]	**TARDIS**	**0.70**	0.68	**0.74**	**0.77**	0.38
Dataset with low-resolution membrane	**8A**	**TARDIS**	**0.46**	**0.61**	**0.37**	**0.61**	**0.18**
	8A [Only Cryo-EM]	TARDIS	0.06	0.48	0.04	0.22	0.03

* high-resolution - Micrograph of resolution smaller or equal to 10 Å

** low-resolution - Micrograph of resolution greater than 10 Å

bioRxiv preprint doi: https://doi.org/10.1101/2024.12.19.629196; this version posted December 20, 2024. The copyright holder for this preprint (which was not certified by peer review) is the author/funder, who has granted bioRxiv a license to display the preprint in perpetuity. It is made available under aCC-BY 4.0 International license.

941 **Supplementary Table 4: Validation of semantic and instance segmentation prediction of**
942 **actin from cryo-ET tomograms**

Type	F1	Precision	Recall	AP	AP90	mCov
**FNet**	**0.07**	**0.58**	**0.04**	**0.54**	**0.06**	**0.12**
UNet	0.06	0.55	0.04	0.50	0.02	0.10

943

bioRxiv preprint doi: https://doi.org/10.1101/2024.12.19.629196; this version posted December 20, 2024. The copyright holder for this preprint (which was not certified by peer review) is the author/funder, who has granted bioRxiv a license to display the preprint in perpetuity. It is made available under aCC-BY 4.0 International license.

Supplementary Table 5: Validation of semantic segmentation prediction of microtubule filaments segmented from tomographic dataset. Precision, recall, and F1 scores are calculated using a probability threshold of 0.5 for TARDIS and Amira.

	Model	F1	Precision	Recall	AP	AP90
All Datasets	**TARDIS [FNet]**	**0.61**	**0.70**	**0.55**	**0.69**	**0.33**
	TARDIS [UNet]	0.60	0.69	0.55	0.27	0.31
Cryo-ET dataset	**TARDIS [FNet]**	**0.58**	**0.72**	0.48	**0.71**	0.20
	TARDIS [UNet]	0.54	0.58	**0.50**	0.70	**0.30**
TEM dataset	**TARDIS [FNet]**	**0.61**	**0.70**	**0.56**	**0.67**	0.35
	TARDIS [UNet]	0.61	0.70	0.55	0.65	**0.37**

bioRxiv preprint doi: https://doi.org/10.1101/2024.12.19.629196; this version posted December 20, 2024. The copyright holder for this preprint (which was not certified by peer review) is the author/funder, who has granted bioRxiv a license to display the preprint in perpetuity. It is made available under aCC-BY 4.0 International license.

Supplementary Table 6: Validation of pixel size normalization on semantic segmentation prediction of membranes from cryo-ET tomograms

Scaling	Type	F1	Precision	Recall	AP	AP90
8A	FNet	0.45	0.78	0.32	**0.69**	0.19
	UNet	0.43	**0.80**	0.29	0.69	0.19
**15A**	**FNet**	**0.66**	0.68	**0.65**	0.58	**0.31**
	UNet	0.66	0.66	0.65	0.57	0.28

bioRxiv preprint doi: https://doi.org/10.1101/2024.12.19.629196; this version posted December 20, 2024. The copyright holder for this preprint (which was not certified by peer review) is the author/funder, who has granted bioRxiv a license to display the preprint in perpetuity. It is made available under aCC-BY 4.0 International license.

Supplementary Table 7: Validation of pixel size normalization on semantic segmentation prediction of membranes from cryo-EM micrographs

	Scaling	Type	F1	Precision	Recall	AP	AP90
All Dataset	**4A**	**FNet**	**0.56**	0.62	**0.55**	0.61	**0.37**
		UNet	0.54	0.64	0.51	0.61	0.37
	8A	FNet	0.51	**0.71**	0.44	**0.68**	0.37
		UNet	0.49	0.67	0.43	0.64	0.34
	15A	FNet	0.46	0.68	0.41	0.66	0.33
		UNet	0.38	0.63	0.31	0.61	0.26
Dataset with high-resolution membrane	**4A**	**FNet**	**0.64**	0.73	**0.62**	0.71	**0.52**
		UNet	0.62	0.74	0.58	0.71	0.52
	8A	FNet	0.54	**0.76**	0.48	**0.72**	0.48
		UNet	0.52	0.70	0.48	0.67	0.48
	15A	FNet	0.47	0.73	0.44	0.69	0.44
		UNet	0.35	0.62	0.28	0.59	0.30
Dataset with low-resolution membrane	4A	FNet	0.41	0.44	**0.42**	0.44	0.11
		UNet	0.40	0.47	0.37	0.45	0.10
	**8A**	**FNet**	**0.46**	0.61	0.37	0.61	0.18
		UNet	0.44	0.60	0.35	0.59	0.14
	15A	FNet	0.44	0.60	0.35	0.59	0.14
		UNet	0.44	**0.64**	0.35	**0.63**	**0.20**

* high-resolution - Micrograph of resolution smaller or equal to 10 Å

** low-resolution - Micrograph of resolution greater than 10 Å

bioRxiv preprint doi: https://doi.org/10.1101/2024.12.19.629196; this version posted December 20, 2024. The copyright holder for this preprint (which was not certified by peer review) is the author/funder, who has granted bioRxiv a license to display the preprint in perpetuity. It is made available under aCC-BY 4.0 International license.

Algorithms, Program Codes, and Listings

Equation 1. F1 score

$$F1 = 2 * \frac{Precision * Recall}{Precision + Recall}$$

Equation 2. Precision score

$$Precision = \frac{Precision * Recall}{Precision + Recall}$$

Equation 3. Recall score

$$Recall = \frac{TP}{TP + FN}$$

Where TP denotes the number of true positive predictions, and FN denotes false negatives.

Equation 4. AP score and AP90

$$AP = \sum_{k=1}^{n} Precision_k * \Delta\, Recall_k$$

Where n is the number of thresholds, and k is the threshold value between 0 and 1. The $Precision_k$ denotes precision at threshold k and $\Delta Recall_k$ denotes a change in recall between k and k-1.

$$AP90 = Precision_k * \Delta\, Recall_k$$

Where k denotes Precision at the threshold where recall is equal to 90%, and $\Delta Recall_k$ denotes a change in recall between k and k-1.

bioRxiv preprint doi: https://doi.org/10.1101/2024.12.19.629196; this version posted December 20, 2024. The copyright holder for this preprint (which was not certified by peer review) is the author/funder, who has granted bioRxiv a license to display the preprint in perpetuity. It is made available under aCC-BY 4.0 International license.

Equation 5. mCov score

$$mCov(I,P) = \frac{1}{|I|}\sum_{m=1}^{|I|} max_n IoU(I_m, P_n)$$

Where I_m denotes the number of projected spline or volume for the m-th ground truth instance. P_m represents the n-th predicted instance, and |I| is the number of all ground truth instances.
